# α/β-Hydrolase Domain (ABHD) Inhibitors as New Potential Therapeutic Options against Lipid-Related Diseases

**DOI:** 10.1021/acs.jmedchem.1c00624

**Published:** 2021-07-02

**Authors:** Giulia Bononi, Tiziano Tuccinardi, Flavio Rizzolio, Carlotta Granchi

**Affiliations:** † Department of Pharmacy, University of Pisa, Via Bonanno 6, 56126 Pisa, Italy; ‡ Pathology Unit, Centro di Riferimento Oncologico di Aviano (CRO) IRCCS, 33081 Aviano, Italy; § Department of Molecular Sciences and Nanosystems, Ca’ Foscari University, 30123 Venezia, Italy

## Abstract

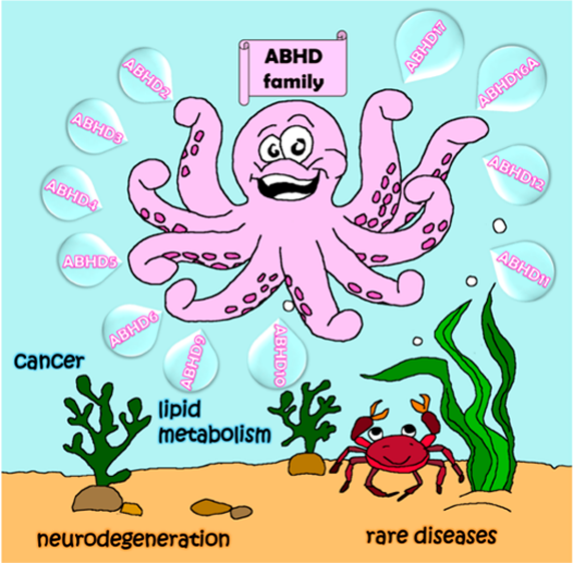

Much of the experimental evidence in the literature has linked altered lipid metabolism to severe diseases such as cancer, obesity, cardiovascular pathologies, diabetes, and neurodegenerative diseases. Therefore, targeting key effectors of the dysregulated lipid metabolism may represent an effective strategy to counteract these pathological conditions. In this context, α/β-hydrolase domain (ABHD) enzymes represent an important and diversified family of proteins, which are involved in the complex environment of lipid signaling, metabolism, and regulation. Moreover, some members of the ABHD family play an important role in the endocannabinoid system, being designated to terminate the signaling of the key endocannabinoid regulator 2-arachidonoylglycerol. This Perspective summarizes the research progress in the development of ABHD inhibitors and modulators: design strategies, structure–activity relationships, action mechanisms, and biological studies of the main ABHD ligands will be highlighted.

## 1 Introduction

Endocannabinoids 2-arachidonoylglycerol (2-AG) and *N-*arachidonoylethanolamine (anandamide, AEA) are endogenous lipid molecules activating the two G protein-coupled cannabinoid receptors 1 and 2 (CB1R and CB2R). 2-AG and AEA are produced following stimulation from phospholipid precursors present in the cell membranes and immediately metabolized after their activation of specific signaling pathways by specific lipases.^1^ Therefore, the endocannaboinoid system (ECS) includes also enzymes controlling endocannabinoid levels and the most important is fatty acid amide hydrolase (FAAH), mainly responsible for the hydrolysis of AEA and monoacylglycerol lipase (MAGL), which is designated for 2-AG inactivation.^2^ In this context, it is noteworthy to introduce a family of endocannabinoid-degrading enzymes which is progressively attracting more interest by the scientific community: the α/β-hydrolase domain (ABHD) enzymes. Muccioli et al. provided the first evidence that not only does MAGL hydrolyze 2-AG, since they found that MAGL was not expressed in the mouse microglial cell line, BV-2, but also a 2-AG hydrolyzing activity was present.^3^ In the same year, ABHD6 and ABHD12 were identified by activity-based protein profiling (ABPP).^2^ In particular, 85% of brain 2-AG hydrolase activity can be ascribed to MAGL, and the remaining 15% is mostly performed by ABHD6 and ABHD12 (4% and 9%, respectively).

Besides ABHD6 and ABHD12 which are related to ECS, many other ABHD enzymes have been identified and they showed specific physiological functions as regulators of lipid metabolism and signal transduction. Their association to human diseases of altered lipid metabolism will be explained in detail in the following specific sections.

Importantly, all ABHD enzymes belong to the α/β-hydrolase fold superfamily,^4^ which includes many different hydrolytic enzymes and shares a common three-dimensional feature since members of this family contain eight β-strands with the second antiparallel strand. The β sheets are surrounded on both sides by α helices and loops connecting the eight sheets. Each member of this family derives its hydrolytic activity from a highly conserved catalytic triad, characterized by the sequence: (a) nucleophile residue (serine, cysteine, or aspartate) located in the nucleophilic elbow in the loop following strand β5; (b) acid residue (glutamate or aspartate) after strand β7; (c) histidine residue located after the last β strand. The active site can be covered by a dynamic lid. Most of the ABHD enzymes are also endowed with acyltransferase activity due to the conserved His-XXXX-Asp region (X is any amino acid).^5,6^

It is noteworthy to underline that the α/β-hydrolase fold superfamily is a very large multifaceted protein family which includes more than 50 enzymes possessing different names. Nevertheless, the present Perspective is focused on those members of this superfamily which are usually named ABHD enzymes, with the aim of highlighting the therapeutic potential of this group of proteins.

Despite the fact that ABHD enzymes are attractive targets for novel therapies targeting cancer and metabolic diseases,^7,8^ the research field concerning the development of inhibitors/modulators of these ABHDs is still quite unexplored. A greater interest has been devoted to ABHD6 and ABHD12 inhibitors, due to their involvement in the ECS. In fact, CB1R and CB2R are involved in many physiological and pathological processes; therefore, beneficial effects derive from their modulation. Nevertheless, it is well-known that their direct activation is associated with many drawbacks such as receptor desensitization and abuse potential. For this reason, more recent therapeutic approaches are directed toward their indirect stimulation by the inhibition of endocannabinoid degradation.^9^ While a growing number of selective and potent inhibitors of FAAH and MAGL have been published or patented in the last decades, the discovered ABHD6 and ABHD12 inhibitors are still in their beginning, since the amount of inhibitors is limited and few of them have been the object of extensive studies.

Many developed ABHD inhibitors reported in the literature and reviewed here were characterized by activity-based protein profiling (ABPP), because ABPP is a proteomic technology used to determine not only the activity in cells and tissues but also the selectivity of ABHD inhibitors in an unbiased proteome-wide fashion. A variety of applications of ABPP have been developed in the last decades, since ABPP combines different scientific disciplines. In order to speed up the drug discovery process, ABPP is able to test inhibitors against many enzymes in parallel, and thus, potency and selectivity can be determined in a saving-time approach.^10^ ABPP relies on the design of small-molecule probes that covalently label the active site of families of enzymes in complex proteomes. In particular, these probes possess (a) a “warhead” that is a chemical portion targeting conserved structural features present in active sites of an enzyme family, such as electrophilic groups binding conserved active-site nucleophile serine of serine hydrolase enzymes, and (b) a reporter tag, to facilitate target characterization, i.e., fluorophores, biotin, and alkynes or azides (which can be modified by Huisgen 1,3-dipolar cycloaddition).^11^ Experimental read-out techniques such as gel-based methods or LC-MS approaches are usually adopted for analyzing probe-treated proteomes.

**Figure 1:**
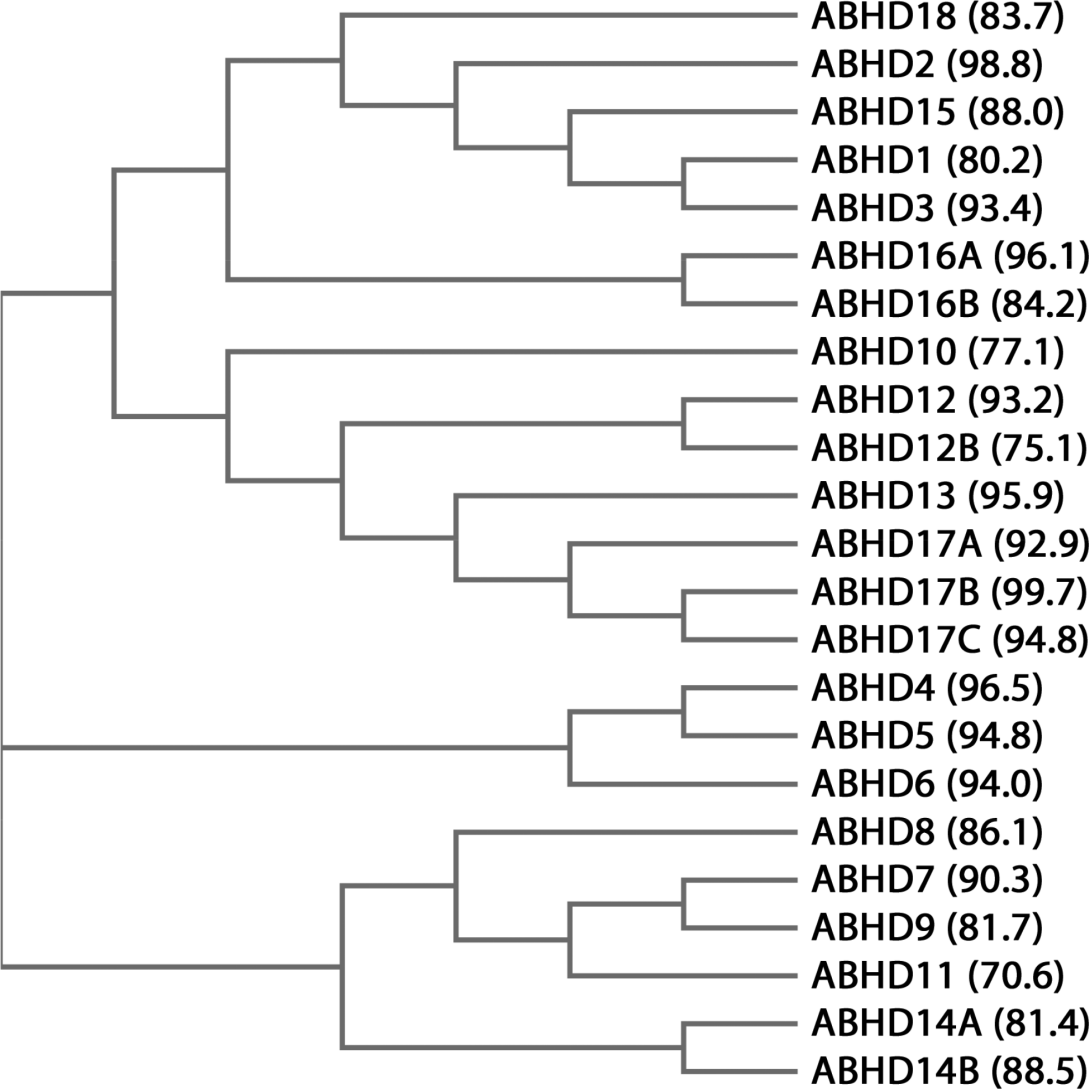
Phylogenetic relationship of the human ABHD proteins. For each protein, the percentage of residue identity between human and mouse species is highlighted between brackets.

All ABHD proteins are reviewed in Table 1 and Figure 1, in which their main features are summarized. In this Perspective, inhibitors and modulators of ABHDs will be reviewed, classifying the compounds on the basis of the specific inhibited ABHD enzyme and on the different chemical families, with a special focus on the specific roles of each ABHD enzyme. Additionally, specific attention will be dedicated to the patented ligands of ABHDs, in particular to those that have not been reviewed elsewhere as dual MAGL/ABHDs inhibitors.^12,13^

**Table 1:** Overview of ABHD Proteins: Main Expression Pattern in Humans and Substrates of Each Protein Are Reported

ABHD protein	main expression pattern	main substrates
ABHD1	testis	–a
ABHD2	ubiquitous expression, liver, stomach	triacylglycerols, esters
ABHD3	appendix, colon, gall bladder, lymph nodes, stomach, thyroid, small intestine, duodenum	medium-chain phospholipids, phosphatidylcholines containing C14 acyl chain, oxidatively truncated phospholipids
ABHD4	testis, gall bladder	–a
ABHD5	bone marrow, fat, skin	arachidonoyl-CoA, oleoyl-CoA, 1-oleoyl-lysophosphatidic, triacylglycerols
ABHD6	small intestine, duodenum, spleen, brain, brown adipose tissue, kidney, liver, skin, ovary	diacylglycerols, 1(3)-monoacylglycerols with saturated medium or long acyl chains, 2-arachidonoylglycerol, lysophosphatidylinositols, bis(monoacylglycero)phosphate
ABHD7	brain	–a
ABHD8	brain, testis	–a
ABHD9	skin, esophagus	epoxyeicosatrienoic acids, 9,10-epoxyoctadecamonoenoic acids, leukotoxin, linoleate-derived epoxy-alcohols
ABHD10	kidney, thyroid	*S*-palmitoyl substrates
ABHD11	skeletal muscle, colon, prostate, small intestine, thyroid	triacylglycerols, 2-oxoglutarate
ABHD12	ubiquitous expression, brain	2-arachidonoylglycerol, 1(3)-isomer of arachidonoylglycerol, unsaturated C20:4 monoacylglycerols, lysophosphatidylserine lipids
ABHD12B	skin	–a
ABHD13	ubiquitous expression	–a
ABHD14A	adrenal glands, brain, kidney, thyroid	–a
ABHD14B	ubiquitous expression	*p*-nitrophenyl butyrate
ABHD15	fat, liver	–a
ABHD16A	ubiquitous expression, skeletal muscle, brain and platelets	medium-chain saturated monoacylglycerols, 1-linoleylglycerol, 15-deoxy-Δ^12,14^-prostaglandin J2-2-glycerol ester
ABHD16B	testis	–a
ABHD17A	bone marrow, fat, lung, skin, spleen	*S*-palmitoyl-l-cysteine residue
ABHD17B	brain	*S*-palmitoyl-l-cysteine residue
ABHD17C	colon, esophagus, stomach, small intestine, brain, duodenum, lung, prostate, urinary bladder	*S*-palmitoyl-l-cysteine residue
ABHD18	ubiquitous expression	–a

a Not determined.

## 2 ABHD2

### 2.1 Biochemical Features and Biological Roles

The serine hydrolase ABHD2 is a 425-residue protein (48 kDa) possessing a typical Ser207-His376-Asp345 catalytic triad, and it is ubiquitously expressed, mainly in liver and stomach. ABHD2 is considered a triacylglycerol lipase and an ester hydrolase.^14^ It is overexpressed in human androgen-sensitive prostate cancer tissues since lipid metabolism plays a key role in the development and progression of this type of tumor. Moreover, high ABHD2 expression is correlated with resistance to docetaxel-based chemotherapy.^15^ Deletion of the ABHD2 gene was correlated to anoikis resistance in high-grade serous ovarian cancer (HGSOC), thus promoting a malignant phenotype and poor prognosis.^16^ Furthermore, ABHD2 was shown to be involved in many diseases such as Hepatitis B virus propagation,^17^ since its downregulation using antisense oligonucleotides blocked Hepatitis B virus replication and expression without affecting host cell physiology. ABHD2 plays a key role in monocyte/macrophage recruitment, therefore influencing the development of chronic diseases such as atherosclerosis and emphysema. In particular, ABHD2 deficiency induced emphysema, due to increased macrophage infiltration, increased inflammatory cytokines and enhanced apoptosis because ABHD2 is important to maintain lung structural integrity.^18^ With regard to its involvement in the pathogenesis of atherosclerosis, ABHD2 genetic deficiency enhances the migration of vascular smooth muscle cells, which is one of the causes of this vascular disease.^19^ In addition, ABHD2 expression was significantly increased in parallel with the differentiation from monocyte into macrophage, and macrophages of atherosclerotic lesions abundantly expressed ABHD2.^20^ High expression of ABHD2 in spermatozoa revealed the ability of this protein to bind progesterone, triggering 2-AG degradation, thus revealing that progesterone-mediated activation of ABHD2 finally stimulates sperm activation.^21^ The same mechanism was induced by pregnenolone sulfate: similarly to progesterone, it activated calcium channel of sperm by ABHD2 binding.^22^ Finally, ABHD2 proved to be involved in the regulation of calcium release from the endoplasmic reticulum (ER).^23^

### 2.2 Inhibitors

**Figure 2:**
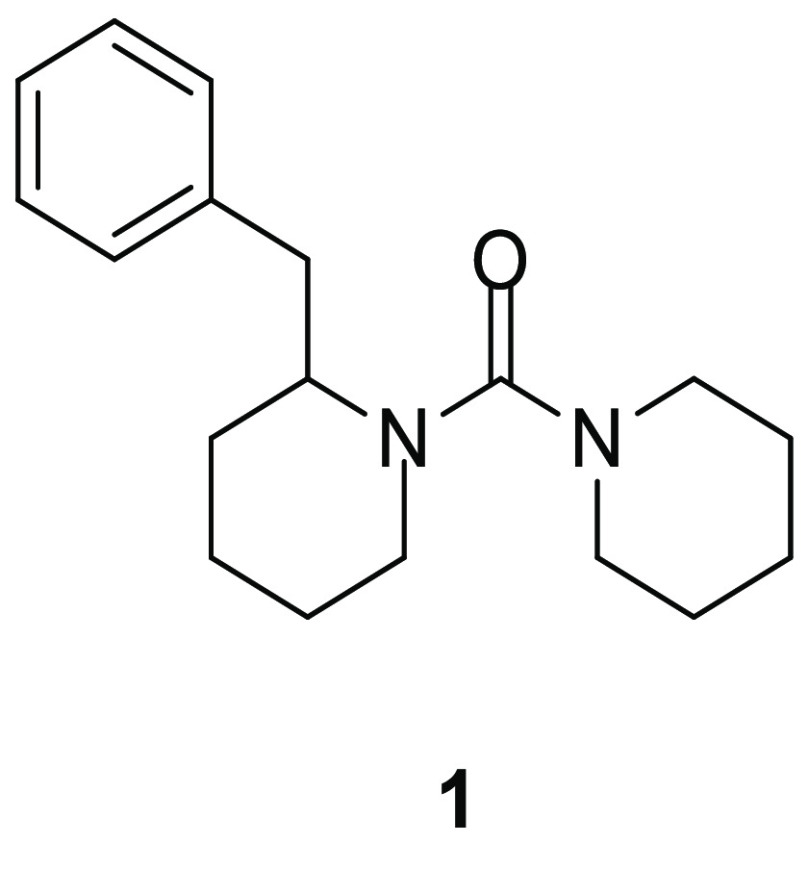
ABHD2 inhibitor.

Very recently, Baggelaar and his research group conducted an ABPP screening based on ABHD proteins and a library composed of 207 lipase inhibitors to identify selective ABHD2 inhibitors.^24^ Urea derivative **1** (Figure 2) exerted a notable activity on ABHD2 (pIC_50_ = 5.50) with no other off-targets in mouse testis proteome. This selectivity assay was performed in this specific proteome, since ABHD2 has an important role in sperm fertility. In order to analyze this aspect, inhibitor **1** was evaluated for its capacity to reduce progesterone-induced acrosome reaction (AR) in vitro, which is an important calcium-dependent process for the fertilization of mammalian eggs by spermatozoa, and it is stimulated by many molecules including progesterone. Compound **1** reduced progesterone-induced AR in vitro in a concentration-dependent manner, by blocking calcium increase induced by progesterone, thus confirming that ABHD2 finely tunes intracellular calcium levels in mouse sperm. These results suggest that urea derivative **1** could represent an interesting starting compound to develop new ABHD2 inhibitors as perspective novel contraceptives.

## 3 ABHD3

### 3.1 Biochemical Features and Biological Roles

ABHD3, previously known as lung α/β-hydrolase 3 (LABH3),^25^ is a poorly characterized 409-residue (46 kDa) serine hydrolase highly expressed in appendix, colon, gall bladder, lymph nodes, stomach, thyroid, small intestine, duodenum, whose biochemical or physiological functions are still scarcely known. ABHD3 showed a multifaceted role in the catabolism of medium-chain phospholipids, that is distinct from those of other known phospholipases, as demonstrated in metabolomic studies.^26^ In fact, ABHD3 showed a good specificity toward phosphatidylcholines (PCs) containing C14 acyl chain and oxidatively truncated phospholipids over other phospholipids. ABHD3 was shown to be upregulated in a series of pathological conditions: in human ovarian cancer cell lines exposed to standard chemotherapeutic drugs (cisplatin, paclitaxel or topotecan),^27^ in breast cancer tumors, as a pro-apoptotic gene,^28^ in a human osteosarcoma cell line overexpressing the tumor suppressor gene HIC1 (Hypermethylated in Cancer 1)^29^ and in mice hippocampus after low-intensity exercise alone and/or in combination with the natural antioxidant carotenoid astaxanthin, revealing an antioxidant function of ABHD3.^30^ Conversely, ABHD3 is downregulated in peripheral blood mononuclear cells of patients affected by Crohn’s disease^31^ and in a rat model of glaucoma characterized by early optic nerve head, which is the principal site of initial axonal injury.^32^

### 3.2 Inhibitors

**Figure 3:**
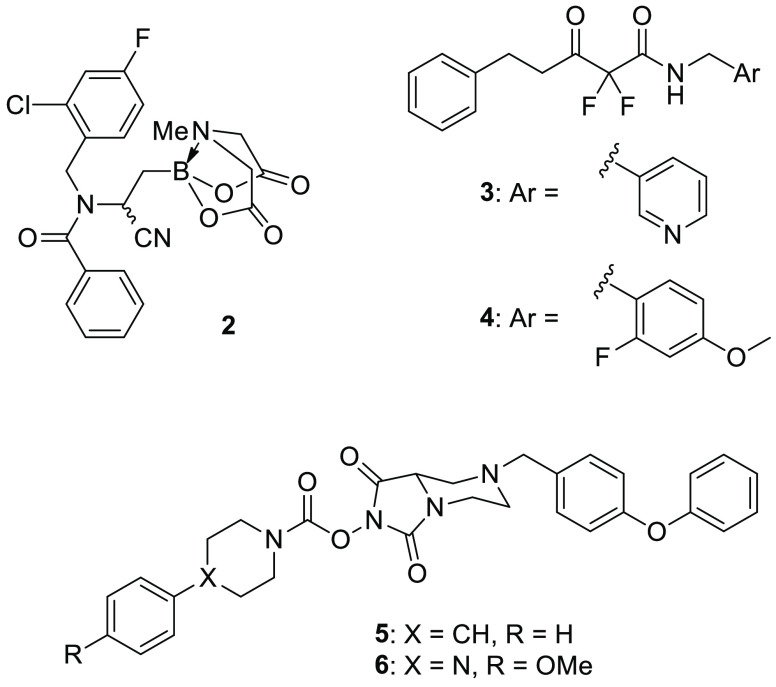
ABHD3 inhibitors.

Tan and collaborators performed a competitive ABPP screening on a library of synthesized α- and β-aminocyano *N*-methyliminodiacetic acid-containing (MIDA) boronates in mouse brain proteome.^33^ Several compounds belonging to this class exhibited ABHD3 inhibition, but further studies on HEK293T (human embryonic kidney cells) lysates overexpressing ABHD3 showed that the most active and selective ABHD3 inhibitor was β-aminocyano(MIDA)boronate **2** (Figure 3), with an IC_50_ value of 0.14 μM in vitro. With regard to **2** selectivity, SDS-PAGE analysis of tissue proteomes was able to identify only a limited number of serine hydrolases. Consequently, the authors further investigated the selectivity of **2** by MS-based ABPP using stable isotope labeling with amino acids in cell culture (SILAC). This technique allowed to confirm the selectivity of boronate **2** on ABHD3 (>95% of blockade at 0.5 μM) without detecting any activity over 60 additional serine hydrolases in human colon cancer cell line SW620. A structure–activity relationship analysis revealed the importance of the phenylamide portion, the cyano group, and the fluorine atom of **2** for inhibition potency. Importantly, the boron atom is fundamental for ABHD3 covalent inhibition, and the MIDA boronate portion seemed to increase cell permeability or stability in cells when compared to the free boronic acid analogue, proving to be resistant to hydrolytic cleavage under neutral conditions during the ABPP experiments. Metabolomic studies of **2** confirmed the previous findings that ABHD3 inhibition leads to an increase of medium-chain PCs in human cells.

It is also noteworthy to cite 1,3-dicarbonyl derivatives **3** and **4** (Figure 3) identified in the previously mentioned screening aimed at finding new ABHD2 inhibitors:^24^ these two compounds proved to selectively inhibit ABHD3 over the other tested ABHD enzymes.

The research group of Cravatt synthesized a small library of *N*-hydroxyhydantoin carbamates and screened them through competitive ABPP on serine hydrolases.^34^ Compounds **5** (ABC47, Figure 3) and **6** (ABC34, Figure 3) demonstrated a good activity on ABHD3 (IC_50_ = 0.1 and 7.6 μM, respectively), but a more potent inhibition was observed on ABHD4 (IC_50_ = 0.03 and 0.1 μM, respectively) in mouse brain membrane proteome. However, ABPP-SILAC experiments in human PC3 cells highlighted that **5** and **6** inhibited not only ABHD3 and ABHD4, but they also had four additional off-targets: ABHD6, hormone-sensitive lipase (HSL), phospholipase A2 Group VII (PLA2G7), and carboxylesterase 2 (CES2). This study suggests that the *N*-hydroxyhydantoin carbamate scaffold could be finely optimized to achieve the inhibition activity toward the desired serine hydrolase.

## 4 ABHD4

### 4.1 Biochemical Features and Biological Roles

Human ABHD4 is composed of 342 residues (39 kDa) and is prevalently expressed in testis and gall bladder. ABHD4 is a lysophospholipase/phospholipase B first identified in 2006 as the enzyme responsible for the deacylation of *N*-acyl phosphatidylethanolamines and lyso-*N*-acyl phosphatidylethanolamines to generate glycerophospho-*N*-acyl ethanolamines, which are intermediates for the biosynthesis of *N*-acyl ethanolamines, an important group of signaling lipids including anandamide.^35^ Later, biochemical and in vivo studies revealed that brain *N*-acyl lysophosphatidylserines are also substrates of ABHD4.^36^ ABHD4 has a beneficial role in a fibrosarcoma model, limiting cell proliferation.^37^ ABHD4 is a regulator of anoikis, which is a programmed cell death of anchorage-dependent cells when they detach from the extracellular matrix, and resistance to anoikis usually leads to cancer metastases. Genetic deletion of ABHD4 induced anoikis resistance in prostate cells as well as nasopharyngeal and ovarian cancer cells; however, the exact mechanism was not yet elucidated.^38^ Very recently, László et al. found that ABHD4 is a necessary mediator for the elimination of pathologically detached cells in embryonic brain, confirming that downregulation of ABHD4 may induce resistance to anoikis.^39^

### 4.2 Inhibitors

**Figure 4:**
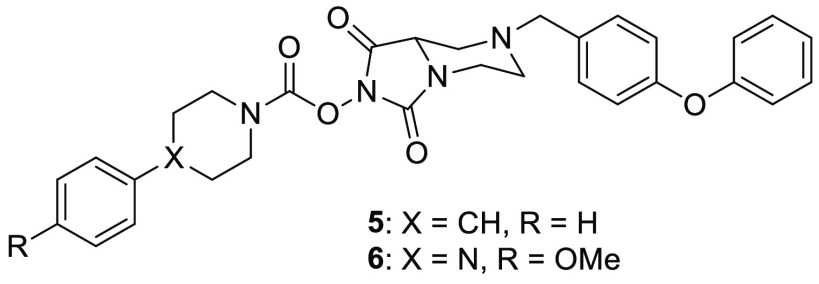
ABHD4 inhibitors.

Very few ABHD4 inhibitors are reported in literature: the most potent are the previously mentioned compounds **5** and **6** (Figure 4) identified by Cognetta et al. These *N*-hydroxyhydantoin carbamates displayed IC_50_ values in the submicromolar range (IC_50_ = 0.03 and 0.1 μM for **5** and **6**, respectively) in mouse brain membrane proteome analyzed by gel-based ABPP, although they are not highly selective for ABHD4, because of their additional inhibition activity on ABHD3 (subsection 3.2). Interestingly, some analogues of compound **6** were further developed as probes for gel-based detection of ABHD4 in ABPP experiments; however, their discussion is out the scope of this perspective. Cognetta et al. identify other ABHD4 ligands, unfortunately none of them were selective for ABHD4 nor reached a greater inhibition potency than compounds **5** and **6**.^34^

## 5 ABHD5

### 5.1 Biochemical Features and Biological Roles

ABHD5 or Comparative Gene Identification 58 (CGI-58) is a well characterized member of this class of ABHDs. It is a 349-residue protein (39 kDa) mainly expressed in bone marrow, fat, and skin. The mutation of ABHD5 gene causes the human Chanarin-Dorfman Syndrome or Neutral Lipid Storage Disease with Ichthyosis (NLSDI), which is a rare autosomal recessive disorder characterized by the presence of intracellular accumulation of triacylglycerol (TG) droplets in many tissues. Multiple organs and tissues are affected by this syndrome, since patients suffering of NLSDI manifest ichthyosis and sometimes liver steatosis with hepatomegaly, muscle weakness (or myopathy), ataxia, neurosensory hearing loss, subcapsular cataracts, nystagmus, strabismus, and mental retardation.^40,41^ ABHD5 mutation is also related to a rare heritable form of nonalcoholic fatty liver disease (NAFLD), a severe health disease associated with significant morbidity and mortality.^42,43^ In ABHD5, the nucleophilic serine is substituted by asparagine; therefore, ABHD5 itself is not able to hydrolyze triacylglycerols, but it coactivates adipose triglyceride lipase (ATGL), an important TG hydrolase which catalyzes the formation of glycerol and free fatty acids.^44^ Mutations in both ATGL and ABHD5 cause the “neutral lipid storage disease” characterized by massive accumulation of TG in various tissues. Knockout of ABHD5 in mice resulted in an excessive lipid storage due to defective activation of ATGL-mediated TG hydrolysis. In fact, newborn mice showed a condition similar to human NLSDI, with severe hepatic steatosis and a defective skin permeability barrier. These studies have highlighted that ABHD5 exhibits a crucial role in cellular TG catabolism by its regulation on ATGL activity.^45^ Differently, the use of antisense oligonucleotides to inhibit ABHD5 expression in adult mice induced severe hepatic steatosis, but at the same time prevented high-fat diet-induced obesity and insulin resistance.^46^ Conversely, when mice were genetically deprived of ATGL, they showed a massive accumulation of lipids in several tissues and the inability to mobilize these fat stores, along with an increase in insulin sensitivity, glucose use, and tolerance.^47^ A further study confirmed that ABHD5 knockdown by antisense oligonucleotides paradoxically improved hepatic insulin signaling, reducing diet-induced stress kinase activation, thus highlighting an important role of ABHD5 in mediating inflammatory responses.^48^ ABHD5 overexpression in mice did not prevent the development of diet-induced obesity; therefore, the ATGL activation induced by ABHD5 is not a determining factor for lipolysis.^49^ Therefore, despite the involvement of both ABHD5 and ATGL in TG hydrolysis, experimental evidence suggests distinct roles of these two proteins.^50^ ABHD5 displayed acyl-CoA-dependent acyltransferase activity to lysophosphatidic acid, showing a preference for unsaturated species of acyl-CoA, such as arachidonoyl-CoA, oleoyl-CoA, and 1-oleoyl-lysophosphatidic acid.^6^ ABHD5 was found to be located in the lipid droplets in adipocytes, thanks to the interaction with perilipin-1 (PLIN1 or perilipin-A), which is expressed almost exclusively in adipocytes,^51^ and it is designated to the breakdown of TG in lipid droplets via its phosphorylation. A mutation of ABHD5, as in Chanarin-Dorfman syndrome, determines a weakening of the ABHD5 binding to PLIN1, suggesting that the loss of this interaction could induce this syndrome.^52^ Lipolytic stimulation by catecholamines triggers the phosphorylation of PLIN1,^53^ disrupts the complex ABHD5/PLIN1, thus inducing release and translocation of ABHD5 from the lipid droplets surface into the cytosol, enabling it to activate ATGL-mediated lipolysis.^54^ The structure of C-terminal moiety of PLIN1 is of crucial importance, because mutations affecting this region proved to make PLIN1 unable to sequester ABHD5, thus triggering ATGL activation and resulting in increased basal lipolysis.^55^ Another isoform of this protein, perilipin-5 (PLIN5 or Mldp), is highly expressed in tissues characterized by high rates of fatty acid oxidation, such as heart, skeletal muscle, and liver, and PLIN5 was able to bind both ABHD5 and ATGL, but not both the protein at the same time.^56^ Both PLIN5 and ABHD5 were observed on the surface of cardiomyocyte lipid droplets, and their interaction was promoted by lipid loading.^57^ Cardiac PLIN5 overexpression regulated ATGL-mediated TG catabolism under regulation of protein kinase A, but PLIN5 does not constantly impair cardiac lipolysis.^58^ Patatin Like Phospholipase Domain Containing 3 (PNPLA3, also known as adiponutrin) interacts with ABHD5 competing with ATGL, so preventing its activation and their binding was much stronger than the interaction of ABHD5 with ATGL. Importantly, PNPLA3 suppressed ABHD5-dependent lipolysis in brown adipocytes.^59,60^ ABHD5 is involved in cancer development: its reduced expression was detected in metastatic castration-resistant prostate cancer and colorectal tumors, in which ABHD5 deficiency induced epithelial to mesenchymal transition and promoted Warburg effect; thus ABHD5 acts as a tumor suppressor.^61,62^ Differently, ABHD5 expression was increased in tumor-associated macrophages in colorectal cancer, and ABHD5 facilitated cancer growth by suppression of spermidine synthase-dependent spermidine production, since spermidine exerts an inhibitory effect on the growth of colorectal cells.^63^ Later, the same authors proved that ABHD5 expressed in macrophages displayed an antimetastatic effect mediated by matrix metalloproteinases, and this opposite finding was justified by the observation that tumor-associated macrophages exhibited heterogeneous expression of ABHD5 and that subgroup of macrophages with low ABHD5 expression was found to be correlated with the invasive behavior of the tumor.^64^ However, the role of ABHD5 in tumors is quite controversial: other studies reported that ABHD5 was overexpressed in prostate cancer cells and ABHD5 genetic deletion decreased growth of prostate cancer cells by inducing apoptosis.^65^ Recent studies demonstrated that overexpression of ABHD5 induces cell cycle arrest at the G1 phase and blocks cell proliferation in prostate cancer cells by inhibition of protein synthesis mediated by mTOR complex 1 (mTORC1); therefore, activation of ABHD5 by ligands may represent a promising therapeutic option against cancer.^66^ ABHD5 was found to be overexpressed and exerted a protumorigenic role in endometrial cancer by involving the AKT signaling pathway.^67^ Travers et al. provided the first evidence of serine protease activity of ABHD5. Histone deacetylases (HDACs) act as repressors of cardiomyocyte hypertrophy through association with the pro-hypertrophic transcription factor myocyte enhancer factor-2 (MEF2). Catecholamine-induced stimulation of β-adrenergic receptors leads to activation of protein kinase A, which triggers the cleavage of HDAC4, with the subsequent production of an amino-terminal polypeptide of HDAC4, and ABHD5 was identified as the one responsible for HDAC4 proteolysis. This series of events ultimately ends with the inhibition of MEF2 transcriptional activity, with resulting protective effects in cultured cardiomyocytes and diabetic hearts, in turn identifying a cardioprotective role for ABHD5. In vivo studies confirmed that ABHD5 lacking mice displayed cardiomyopathy typically associated with neutral lipid storage disease.^68,69^

### 5.2 Modulators

**Figure 5:**
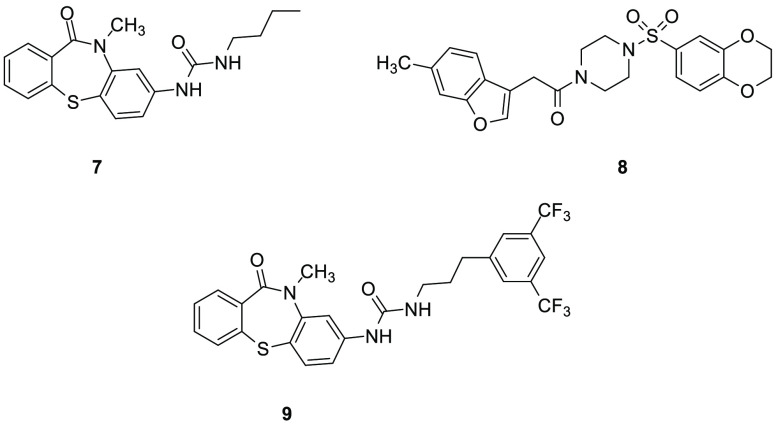
ABHD5 allosteric modulators.

In 2015, Sanders et al. developed the only existing synthetic ABHD5 ligands, which may be useful to target lipid disorders such as obesity, diabetes, and cardiovascular diseases, because of their ability to promote fat catabolism.^70^ The authors considered previous studies assessing that PLIN1 suppresses lipolysis by binding ABHD5, thus preventing ABHD5-mediated activation of ATGL. On the other side, phosphorylation of PLIN1 by protein kinase A led to ABHD5 release, which activates ATGL, thus promoting lipolysis in adipocytes.^53^ High-throughput screening identified two compounds able to disrupt the interaction between ABHD5 and PLIN1 or PLIN5 in the absence of protein kinase A activation: the thiaza-tricyclic urea **7** (SR-4995, Figure 5) and the sulfonyl piperazine **8** (SR-4559, Figure 5). These two derivatives prevented the binding of ABHD5 to PLIN1, with IC_50_ values of 200 and 510 nM, respectively. The newly developed ligands **7** and **8** directly bound to ABHD5 and were shown to be potent and specific allosteric modulators of this enzyme. In brown adipocytes, **7** quickly disrupted the complex between ABHD5 and PLIN5. Inhibitors **7** and **8** were also tested in adipocytes and muscles to evaluate their effects on lipolysis, and they rapidly stimulated lipolysis, displaying EC_50_ values of 4–7 μM. ABHD5 knockdown experiments highlighted that ABHD5 deletion abolished the efficacy of synthetic ligands **7** and **8** of stimulating lipolysis. Moreover, these two compounds promoted dissociation of ABHD5 from PLIN1 or PLIN5, without affecting the ABHD5 capacity to activate ATGL. These two compounds, together with **9** (SR-3420, Figure 5), another thiaza-tricyclic urea derivative differing from **7** only in the presence of the 1,3-(trifluoromethyl)benzene substituent at the end of the alkyl-urea chain, were subjected to further biological experiments.^71^ Compound **9** was more effective in inducing lipolysis than **7** or **8** in white and brown adipocytes. Activation of ABHD5 by **9** potently inhibited mTORC1, thus blocking mTORC1 signaling and inhibiting the anabolism of cancer cells.^66^ Inhibitor **9** regulated the interaction between ABHD5 and PNPLA3 by increasing this interaction.^59^

Compounds **7** and **8** were also the object of a patent dating from 2016, claiming small molecules as modulators of cellular lipolysis.^72^ The authors declared that these modulators, by facilitating fat catabolism, may be used as therapeutic agents to cure diabetes, obesity, cardiovascular diseases but also cancer. Moreover, these derivatives could increase the content of skin barrier lipids upon topical application. Structure–activity relationship (SAR) studies focusing on the thiaza-tricyclic urea scaffold highlighted that the urea at C4 of the tricyclic ring was fundamental for the activity, while the shift at C3 caused a loss of activity; moreover, the replacement of the urea group with esters, amides and *N*-dialkyl ureas at C4 was detrimental for ABHD5 activity. The activity against this enzyme increased by introducing long alkyl chains on the urea group (i.e., *n*-butyl chain in **7**), in particular those ending with aryl groups, while the presence of an oxygen atom in this side chain decreased activity. In addition, small substituents were preferred on the nitrogen atom of the amidic group (i.e., methyl group of **7**). SAR studies on the sulfonyl-piperazine scaffold demonstrated that the length of the linker between the sulfonyl-piperazine moiety in **8** and the benzofuran ring could be slightly increased but this modification decreased the inhibition potency. For what concerns the benzofuran ring, it tolerated alkyl substituents such as methyl group as in **8** and a benzothiazole ring without electron-donating substituents was also allowed. Moreover, if the benzofuran was connected at C2 to the rest of the molecule, the potency was reduced.

## 6 ABHD6

### 6.1 Biochemical Features and Biological Roles

Serine hydrolase ABHD6 is a 337-amino acid protein (38 kDa) with its catalytic triad composed of Ser148-Asp278-His306. It is an integral membrane protein possessing a *N*-terminal transmembrane helix^73^ and is ubiquitously expressed, in particular in brain (cerebellum, frontal cortex, hippocampus, and striatum),^74^ small intestine (duodenum), brown adipose tissue,^75^ spleen, skin liver, kidney, and ovary.^76^ Moreover, female hormones such as estradiol and progesterone can promote the overexpression of ABHD6 in immune cells.^77^ ABHD6 is an important enzyme not only in the central nervous system but also in peripheral tissues, and it is involved in many physiological and pathological states.^78−80^ ABHD6 is significantly expressed in several cancer cell lines, such as bone, prostate, and leukocyte tumor cell lines.^76^ ABHD6 expression is increased in Ewing family tumors (EFT), thus representing a possible diagnostic and/or therapeutic target for this disease, although ABHD6 knockdown in EFT cell lines did not result in a decreased proliferative activity or increased apoptosis rate.^81^ Human pancreatic ductal adenocarcinoma (PDAC) cell lines displayed an high expression of ABHD6, and this enzyme was considered the driving force for the metastatic potential of PDAC cells.^82^ ABHD6 is an important oncogene in non-small-cell lung carcinoma (NSCLC) cells,^83^ since ABHD6 silencing reduced migration and invasion in vitro as well as metastatic potential and tumor growth in vivo. Differently, ABHD6 was identified as an antioncogene in hepatocellular carcinoma (HCC).^84^ A recent study revealed a diacylglycerol lipase (DAGL) activity for ABHD6 in Neuro-2a cells.^85^ A study identified ABHD6 as the main monoacylglycerol lipase present in pancreatic islet β-cells, in which glucose-stimulated insulin secretion is amplified by ABHD6 inhibition. This effect was ascribed to reduced hydrolysis of 1-monoacylglycerols, which activated the protein Munc13-1 (a key exocytotic effector), thus triggering insulin secretion.^86,87^ Deprivation of ABHD6 in mice fed with a high-fat diet induced a reduction of weight gain and liver steatosis, an improved glucose tolerance and insulin sensitivity, an enhanced locomotor activity, and browning of white adipose tissues. In particular, the mechanism of adipose browning behind ABHD6 suppression seems to involve an increase in 1-monoacylglycerols (MAGs), which causes peroxisome proliferator-activated receptors α and γ (PPARα and PPARγ) activation.^88^ A study was focused on the role of ABHD6 in the central control of energy homeostasis. ABHD6 knockdown in neurons of the ventromedial hypothalamus in mice led to impaired adaptive responses to high-fat feeding, dieting, and cold exposure, thus underlining the importance of ABHD6 in maintaining a good flexibility in energetic metabolism.^89^ Some studies highlighted the correlation between ABHD6 expression and the pathogenesis of Epstein–Barr virus (EBV)-related diseases^90^ and the autoimmune disease systemic lupus erythematosus.^91^ As anticipated, the main substrate of ABHD6 is 2-AG:^2,92,93^ ABHD6 controls 2-AG at the site of 2-AG production (postsynaptic), differently MAGL exerts the control at the site of CB1R (presynaptic). The intracellular orientation of ABHD6 is strategic to regulate 2-AG production at the site of its formation. ABHD6 preferentially cleaves MAGs possessing saturated acyl chains, with medium or long chains, with a preference for 1(3)-isomers compared to 2-isomers.^94^ Considering that ABHD6 increases the formation of arachidonic acid by hydrolyzing 2-AG, it is easy to explain its involvement in inflammatory processes. ABHD6 inhibition reduces lipopolysaccharide (LPS)-induced macrophage activation by increasing 2-AG levels in vitro, since 2-AG oxygenation by cyclooxygenase-2 (COX-2) led to the formation of anti-inflammatory prostaglandin D_2_-glycerol ester (PGD_2_-G). ABHD6 was also able to reduce LPS-induced inflammation in mice without provoking the typical central effects of MAGL inhibition^95,96^ (cannabinoid behavioral and functional antagonism of the endocannabinoid system due to chronic MAGL inhibition).^97^ The role of ABHD6 in peripheral tissues was established by Thomas et al. using antisense oligonucleotides to knock down the enzyme in vivo. ABHD6 proved to be implicated in lipid metabolism, since ABHD6 inhibition resulted in the accumulation of lysophosphatidylglycerol (LPG) and phosphatidylglycerol (PG). It exerted a protecting activity from high-fat-diet-induced obesity, hepatic steatosis, hyperglycemia, hyperinsulinemia and it improved both glucose and insulin tolerance in mice. Therefore, ABHD6 contributes to the development of the metabolic syndrome.^75^ ABHD6 is implicated in lysophosphatidylinositols (LPI) metabolism in J774 macrophages as ABHD6 inhibition led to an increase of the levels of all LPI. The effect of ABHD6 inhibition was investigated in LPS-activated J774 cells to study the role of this enzyme in the response of an inflammatory setting. The authors of this study observed an increase in 20:4 LPI levels, therefore ABHD6 could be involved in the hydrolysis of 20:4 LPI; however, extensive studies are still needed to clarify the complex metabolic pathways of LPI.^98^ Bis(monoacylglycero)phosphate (BMP), a phospholipid present in the intraluminal vesicles of late endosomes and lysosomes exerting a fundamental role in degradation and sorting of lipids, was identified as a substrate of ABHD6, thus revealing a role for ABHD6 in the late endosomal/lysosomal lipid sorting.^99^ A more recent study pointed out that ABHD6 affected circulating BMP levels both in mice and humans; consequently deletion of ABHD6 led to increased BMP concentrations without provoking lysosomal storage disorders (LSDs).^100^ These studies suggest that ABHD6 is a key regulator of different classes of lipids. High expression of ABHD6 was detected in an animal model of multiple sclerosis (cuprizone model of nonimmune dependent demyelination) and pharmacological blockade of this hydrolase partially attenuated demyelination and astrogliosis.^101^ The role of ABHD6 was investigated in another animal model of multiple sclerosis, the experimental autoimmune encephalomyelitis (EAE): the use of an ABHD6 inhibitor remarkably ameliorated the clinical signs of EAE, exerting an anti-inflammatory and neuroprotective action.^102^ However, more recently, the therapeutic efficacy of the pharmacological blockade of ABHD6 in improving the clinical signs of EAE was discredited, considering that the ABHD6 inhibition resulted only in a modest slowdown of EAE progression.^103^ Wei and co-workers performed studies regarding the involvement of ABHD6 and α-amino-3-hydroxy-5-methyl-4-isoxazolepropionic acid (AMPA)-type glutamate receptors (AMPARs), which are tetrameric receptors formed by GluA1–4 subunits. ABHD6 inhibited the glutamate-induced currents of GluA1-, GluA2-, and GluA3-containing AMPARs, by binding to GluA1–3 C-terminal regions.^104,105^ Pharmacological ABHD6 inhibition in a mouse model of traumatic brain injury had multiple positive effects: it improved motor coordination and working memory performance due to a reduction of brain lesions, neuroinflammation, neurodegeneration and blood-brain dysfunctions.^106^ An antiepileptic role was reported for ABHD6: ABHD6 pharmacological inhibition reduced pentylenetetrazole-induced seizures and also blocked spontaneous seizures in R6/2 mice, a genetic model of Huntington’s disease characterized by dysregulated endocannabinoid signaling. This study suggests that the observed anticonvulsive effect was independent of cannabinoid receptors, but it involved GABA_A_ receptors; however, further experiments are needed to confirm the above-mentioned mechanism of action.^107^

### 6.2 Inhibitors

#### 6.2.1 Carbamate Derivatives

**Figure 6:**
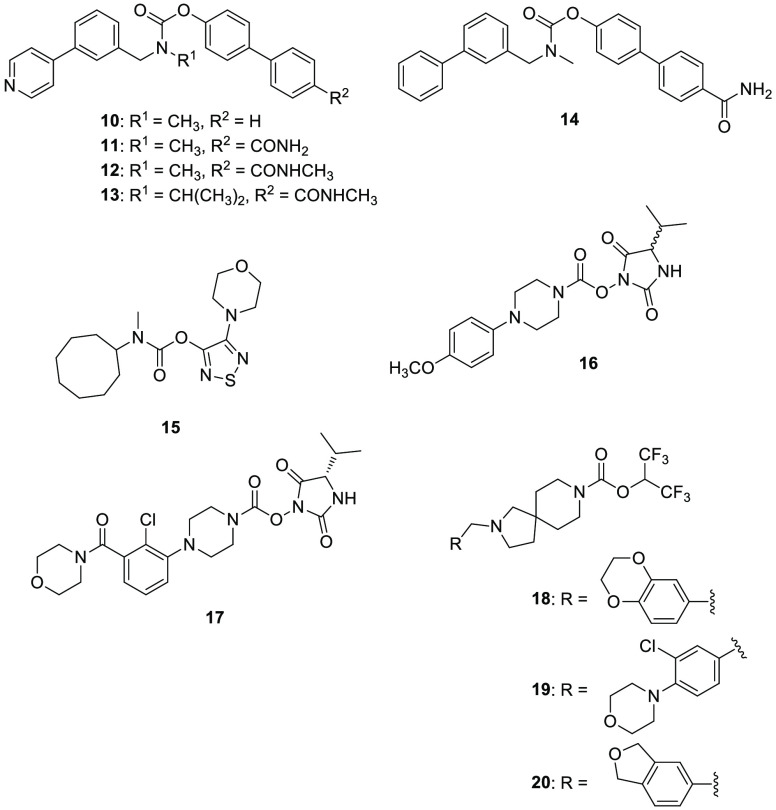
Carbamate-based ABHD6 inhibitors.

Cravatt and collaborators performed a competitive ABPP in COS-7 cells transfected with the human ABHD6 and a library of known carbamate serine hydrolase inhibitors, with the aim of demonstrating that ABPP can be applied for the identification of potent and selective inhibitors for serine hydrolases.^108^ On the basis of this strategy, carbamate **10** (Figure 6) was the most potent and selective inhibitor of this library on ABHD6 (IC_50_ = 350 nM). Compound **10** was further optimized to improve ABHD6 inhibition potency and among the 20 newly synthesized derivatives compound **11** (WWL70, Figure 6), which differs from compound **10** only for the presence of a *p*-carboxamide group in the *para* position on the terminal phenyl ring, showed the highest ABHD6 inhibitory activity, with an IC_50_ value of 70 nM, still maintaining an excellent selectivity. Compound **11** was widely investigated in further pharmacological studies. It inhibited of about 50% the [^3^H]-2-AG hydrolysis in homogenates prepared from neurons in primary culture, whereas the inhibition of [^3^H]-2-AG hydrolysis was reduced to about 20% in homogenates prepared from adult mouse brain, without exerting significant effects in homogenates prepared from microglia in primary culture. These findings are consistent with the fact that ABHD6 activity is greater in neurons in primary culture than in adult mouse brain and ABHD6 expression is very low in microglia in primary culture.^93^

Further pharmacological evaluations highlighted the potential therapeutic role of **11** in animal models of traumatic brain injury and experimental autoimmune encephalomyelitis, as previously described.^102,106^ Kiritoshi et al. tested **11** in an arthritis pain model: **11**, by increasing 2-AG levels and hence activating CB1R, rescued the metabotropic glutamate receptor 5 (mGluR5) activity with a consequent restore of the medial prefrontal cortex output and cognitive function, in addition it reduced pain in the animal model.^109^ Tanaka et al. reported that the anti-inflammatory and neuroprotective properties of **11** were not attributable to ABHD6 inhibition but to its interference with the metabolic pathway from arachidonic acid to prostaglandin E2 (PGE_2_).^110,111^ In particular, derivative **11** blocked PGE_2_ production and the expression of COX-2 and microsomial prostaglandin E synthase-1/2 (mPGES-1/2), the metabolic enzymes necessary for PGE_2_ production from arachidonic acid, in LPS-activated microglia cells and in an animal model of neuropathic pain (chronic constriction injury of the mouse sciatic nerve), thus proving its possible use for the treatment of inflammatory diseases and neuropathic pain.

Madiraju et al. deposited three patents showing that ABHD6 activity is tightly correlated to insulin secretion and to conversion of white into brown adipose tissue.^112−114^ In particular, the three patents described ABHD6 inhibitors which promoted insulin secretion by increasing the accumulation of MAGs and that may be useful for the treatment of type-2 diabetes, insulin resistance and metabolic syndrome. In a cell-based model for insulin secretion, regulation, and pancreatic islet β-cell function studies, the carbamate derivative **11** and the related analogues **12** and **13** (Figure 6) showed 95%, 98%, and 95% of ABHD6 inhibition, respectively, when tested at 10 μM. Moreover, they displayed an increased percentage of insulin secretion compared to control. Finally, compound **11** exerted a benefic effect on mice blood glucose level by increasing plasma insulin concentrations, thus confirming its potential application for treating type-2 diabetes and any other conditions associated with a low level of insulin secretion/production.^113^

A novel carbamate-based compound, **14** (WWL123, Figure 6), was discovered by Cravatt’s research group in 2010 by an ABPP screening.^115^ Compound **14** is a selective ABHD6 inhibitor (IC_50_ = 0.43 μM), which also maintained its selective inhibitory activity on ABHD6 in vivo (mice treated with 5–20 mg/kg, i.p., 4 h). Carbamate **14**, thanks to its high permeability to the blood-brain barrier, exerted an antiepileptic activity in vivo, as previously described.^107^

The 1,2,5-thiadiazole carbamate scaffold, present in potent inhibitors of lysosomal acid lipase, was properly optimized by Patel et al. to develop selective ABHD6 inhibitors, considering that many carbamate-based compounds were found to efficiently inhibit enzymes of the ECS.^116^ The most potent ABHD6 inhibitor of this class was **15** (JZP-430, Figure 6), possessing a carbamate moiety linked to a saturated eight-membered ring in position 3 of the thiadiazole ring and a morpholine ring in position 4 of the central heterocycle, in order to balance the increased lipophilicity determined by the big ring size on the other position. Compound **15** showed an IC_50_ value of 44 nM in lysates of HEK293 cells transiently expressing human ABHD6 and it was also able to inhibit ABHD6 in competitive ABPP of the mouse brain membrane proteome. Derivative **15** was endowed with a good selectivity for ABHD6 over FAAH (only 18% inhibition when tested at 10 μM concentration), and it maintained only a negligible residual activity on lysosomal acid lipase (<20% when tested at 10 μM concentration), without exerting any appreciable activity on cannabinoid receptors, ABHD12 and MAGL. As expected, **15** inhibits ABHD6 by an irreversible mechanism of action. The class of 1,2,5-thiadiazole carbamates was subjected to comparative molecular field analysis (CoMFA) and molecular dynamic (MD) studies on a homology model of ABHD6.^117^ This study highlighted that the most important bond was the hydrogen bond established between the carbonyl group of **15** and the Phe80 backbone, one of the two residues forming the oxyanion hole, thus demonstrating the proper fitting of the compound in this region of the enzyme. During MD simulations, the ABHD6-**15** complex was quite stable; however, the distance between the carbonyl group of the ligand and the Phe80 backbone increased during the simulation, thus weakening the hydrogen bond. On the other hand, the formation of an additional hydrogen bond between Ser148 and the carbonyl group of the inhibitor promoted the covalent bond necessary for the irreversible inhibition of the enzyme.

In the previously mentioned screening of Cognetta et al., *N*-hydroxyhydantoin carbamate **16** (MJN193, Figure 6), characterized by an isopropyl group and a *N*-substituted piperazine on the hydantoin moiety, showed a considerable activity and selectivity for ABHD6.^34^

In 2017, Abide Therapeutics, Inc. patented a series of dual lipoprotein-associated phospholipase A2 (Lp-PLA2) and ABHD6 inhibitors for the treatment of several pathological conditions such as multiple sclerosis, ischemia, traumatic brain injury, Alzheimer’s disease, Parkinson’s disease, amyotrophic lateral sclerosis, cancer, and diabetes.^118^ These newly developed Lp-PLA2/ABHD6 inhibitors show the common chemical structure of 2,5-dioxoimidazolidin-1-yl phenylpiperazine-1-carboxylates, resembling *N*-hydroxyhydantoin **16**. They were tested in vitro (ABPP assays) to evaluate their inhibition potency on both enzymes, and they did not show selectivity for ABHD6. Representative compound **17** (Figure 6) showed an IC_50_ value lower than 100 nM for ABHD6 and between 100 nM and 1 μM for Lp-PLA2.

In the same year, a series of spirocyclic-fused carbamates as modulators of MAGL/ABHD6 was reported by the same company for the treatment of pain.^119^ These dual MAGL/ABHD6 inhibitors were characterized by a hexafluoropropan-2-yl piperidine-1-carboxylate moiety. The most promising derivatives for what concerns ABHD6 inhibition potency were compounds **18**, **19**, and **20** (Figure 6). All the three spirocyclic-fused carbamates proved to be slightly selective for ABHD6 versus MAGL and FAAH. Indeed, they showed IC_50_ values lower than 100 nM on ABHD6, between 100 and 1000 nM on MAGL and greater than 1000 nM on FAAH. Compounds **18**, **19**, and **20** displayed a MAGL and ABHD6 inhibition activity greater than or equal to 75% at 1 μM, on the contrary FAAH inhibition activity was lower than 25% when tested in competitive ABPP assays in mouse brain membrane fraction. No in vivo data were available for these three inhibitors.

#### 6.2.2 Triazole Urea Derivatives

**Figure 7:**
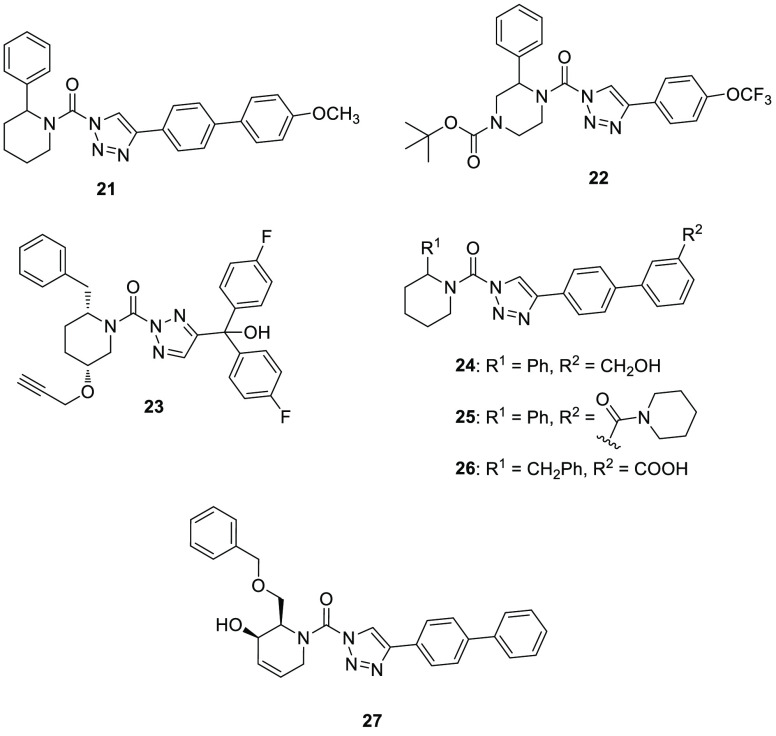
Triazole urea-based ABHD6 inhibitors.

The 1,2,3-triazole urea scaffold is a typical feature of serine hydrolase inhibitors,^120^ in particular in 2012, the research group of Prof. Cravatt focused on this scaffold to develop new DAGL inhibitors. In this screening campaign, the piperidyl-1,2,3-triaziole urea **21** (KT195, Figure 7) was identified as a selective ABHD6 inhibitor (IC_50_ = 10 nM) in competitive ABPP with a marginal cross-reactivity against DAGLβ,^121,122^ and it was predicted to irreversibly bind to the enzyme, by carbamoylating the enzyme’s serine nucleophile. In Neuro-2a cells, **21** confirmed its inhibition activity by fully blocking ABHD6 with an IC_50_ value of 1 nM and a negligible inhibition of DAGLβ. Similarly, in peritoneal macrophages from inhibitor-treated mice, **21** inhibited ABHD6 and lowered interleukin-1β secretion from LPS-treated macrophages; however, two carboxylesterases (CES3 and CES2G) and lysosomal phospholipase A2 group XV (PLA2G15) were identified as off targets of this compound. Compound **21** was further studied to evaluate its potential role to block necrotic cell death.^123^ This ABHD6 inhibitor was able to attenuate necrotic cell death of cultured fibroblasts by preventing mitochondrial calcium uptake and permeability transition pore formation. In addition to the above-mentioned off-targets, **21** also blocked ER calcium release and cell death by targeting the nucleophilic serine in ABHD2.^23^

On the basis of ABHD6 inhibitor **21** and with the aim of obtaining new selective and central nervous system (CNS)-active inhibitors of DAGLα and β, Ogasawara and collaborators synthesized a new triazole urea **22** (DO53, Figure 7), characterized by a 2-phenyl-piperazine moiety instead of the 2-phenyl-piperidine group of **21**.^124^ After intraperitoneal administration to C57BL/6 mice, it showed a good selectivity on ABHD6 together with inhibition of PLA2G7, and a low potency on the original DAGL targets. The selectivity profile of **22** was more extensively elucidated by ABPP experiments coupled to quantitative high-resolution mass spectrometry: it confirmed a negligible activity against DAGLs, but it showed notable cross-reactivity with many other targets, such as ABHD2, ABHD3, carboxylesterase CES1C, and the platelet activating factor acetylhydrolase 2 (PAFAH2). In the same research program, the potent DAGLα and β inhibitor **23** (DH376, Figure 7) showed an undesired ABHD6 inhibition activity both in vitro and in vivo and also a cross-reactivity with carboxylesterase CES1C and HSL.^124,125^ Later, **23** was used to identify the enzymes responsible for 2-AG production during retinoic acid (RA)-induced neurite outgrowth of murine neuroblastoma Neuro-2a cells.^85^ The terminal alkyne group present in the chemical structure of **23** was used in a “click chemistry” approach to introduce reporter tags, which allowed one to visualize by a chemical proteomic strategy the targets of **23** in Neuro-2a cells. ABHD6 and DAGLβ were identified as the only targets and ABHD6 was found to hydrolyze diacylglycerols, thus contributing to the production of 2-AG during RA-induced differentiation of Neuro-2a cells, since **23** blocked 2-AG production and reduced neuronal differentiation.

Compound **21** was structurally optimized by Hsu et al. to improve its potency, selectivity and in vivo activity toward ABHD6. In this new series of irreversible piperidyl-1,2,3-triazole urea inhibitors, compounds **24** (KT182, Figure 7), **25** (KT185, Figure 7), and **26** (KT203, Figure 7) showed a remarkable inhibitory activity against ABHD6, with IC_50_ values of 1.7, 1.3, and 0.82 nM, respectively, corresponding to 0.24, 0.21, and 0.31 nM, when their potencies were measured in situ in Neuro-2a cells. None of them exerted any significant off-target activity.^126^ In these compounds, polar substituents were added in *meta* position of the biphenyl moiety (R^2^ group, Figure 5), such as hydroxymethyl (**24**), piperidine-amide (**25**), or carboxylic acid (**26**). The quantitative mass-spectrometry-based proteomic method ABBP-SILAC was applied to verify their activities: both **24** and **26** inhibited >90% of ABHD6 activity, while **24** blocked >80% of ABHD6 activity in Neuro-2a cells. In addition, the three developed inhibitors did not show any considerable cross-reactivity toward a panel of serine hydrolases present in Neuro-2a cell line, confirming their selectivity for ABHD6 in living cells. Compounds **24** and **26** were also tested in vivo when intraperitoneally administered in mice: both compounds were effective in blocking ABHD6 in the liver at the higher tested dose (1 mg/kg), and only **24** reached the same effect in the brain, probably due to the carboxylic acid of **26** which hinders its brain penetration. A mild systemic inhibitory effect on a plasma esterase carboxylesterase-1 (CES1) was detected only for **24**. Encouraging results were observed with compound **25**, that proved to be an orally bioavailable and selective ABHD6 inhibitor in vivo, even if complete ABHD6 inhibition was only observed at higher dose (40 mg/kg). As anticipated in subsection 6.1, Manterola and co-workers used **24** in the cuprizone model of nonimmune dependent demyelination,^101^ because of its ability to cross the blood-brain barrier and its selectivity in vivo after intraperitoneal administration. After the promising results of this first evaluation, the use of ABHD6 inhibitors was reassessed in multiple sclerosis by testing compounds **24** and **26**, showing different CNS permeability.^103^ The administration of systemically active inhibitor **24** modestly attenuated the neurological disability of the EAE; on the contrary, the peripherally active inhibitor **26** was not effective in ameliorating the clinical signs of EAE. Both compounds **24** and **26** did not attenuate inflammatory responses associated with tissue damage in the chronic phase of EAE, and the chronic treatment with **24** caused the desensitization of brain CB1R. All together, these results suggest that ABHD6 blockade has only a moderate therapeutic effect in this model of demyelination.

A series of dual ABHD6 and DAGLα inhibitors were recently published by Deng et al.^127^ Their strategy aimed at finding dual inhibitors as potential therapeutic agents to treat metabolic and neurodegenerative diseases. This series of dual inhibitors bear the chiral hydroxylated 2-benzylpiperdine scaffold with a triazole urea moiety. Surprisingly, some of them including compound **27** (Figure 7), showed a good combination of inhibition activity of ABHD6 (pIC_50_ = 6.6 in membranes from HEK293T cells expressing recombinant human ABHD6; 83% inhibition in ABPP experiments) and selectivity for ABHD6 versus DAGLα (4-fold) and other serine hydrolases such as FAAH and MAGL.

In conclusion, 1,2,3-triazole urea represents a suitable scaffold to design irreversible inhibitors of serine hydrolases, thanks to the electrophilic carbonyl group which promotes the binding to the nucleophile active site serine. To date, all DAGL inhibitors reported in literature also inhibit ABHD6,^128^ and this aspect may be exploited as the starting point to develop new selective ABHD6 inhibitors, by conveniently modifying this chemotype. Moreover, some activity-based probes (i.e., compounds binding to the enzyme covalently, useful to detect the amount of the enzyme present in a biological system) based on the triazole-urea scaffold were developed to target ABHD6, thus highlighting the high versatility of this chemical core.^129^

#### 6.2.3 Other ABHD6 Inhibitors

**Figure 8:**
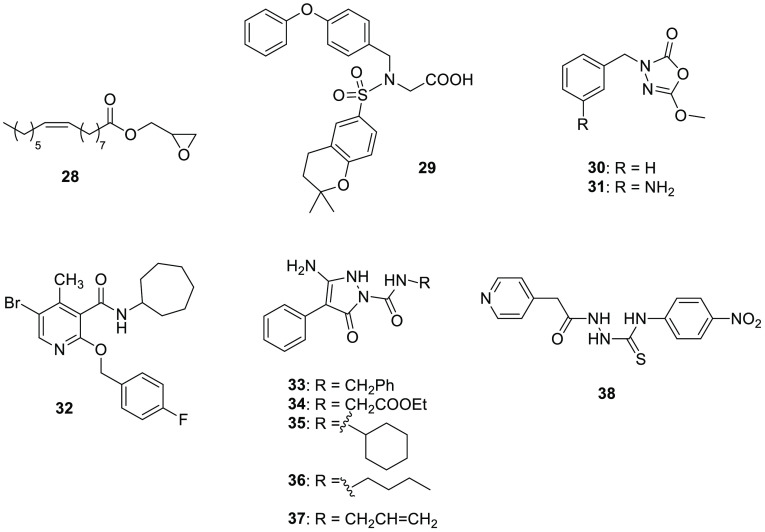
Various ABHD6 inhibitors.

In 2011, Marrs and co-workers designed a series of esters by replacing the glycerol polar head of 2-AG with various oxygenated heterocycles.^130^ The ester derivative **28** (UCM710, Figure 8), characterized by an oxirane moiety, proved to be a potent dual inhibitor of ABHD6/FAAH (IC_50_ values of 2.4 and 4.0 μM, respectively) when tested in neuron homogenates, without inhibiting MAGL nor binding to cannabinoid receptors. Additionally, it was able to efficiently inhibit 2-AG and EAE hydrolysis also in intact neurons, although without reaching the maximum activity (60% and 30% inhibition of AEA and 2-AG hydrolysis, respectively). The unique pharmacological profile of **28** may be determined by its chemical structure, which mimics the natural substrates of the target enzymes, thus likely the oxirane group cannot fit into the active site of MAGL which is covered by the cap domain, differently from ABHD6 and FAAH that lack the cap domain necessary for the substrate recognition and interaction.

In 2014, Janssen and collaborators developed a series of glycine sulfonamides as novel DAGLα inhibitors. A member of this chemical class, compound **29** (LEI106, Figure 8), acts as a submicromolar dual ABHD6/DAGLα inhibitor.^131^ In the colorimetric biochemical assay performed in HEK293 membranes overexpressing human DAGLα, the sulfonamide **29** showed an IC_50_ value of 18 nM and inhibited the hydrolysis of DAGLα natural substrate, [^14^C]-*sn*-1-oleoyl-2-arachidonoyl-glycerol, with a *K*_i_ value of 0.7 μM. After observing an off target in brain membrane homogenate-based assays, a specific biochemical human ABHD6 activity assay revealed that **29** inhibited ABHD6 with a *K*_i_ value of 0.8 μM.

The 1,3,4-oxadiazol-2-one scaffold is widely adopted for the discovery of serine hydrolase inhibitors. Patel et al. optimized the 1,3,4-oxadiazol-2-one **30** (Figure 8),^132^ in order to develop new potent and selective ABHD6 inhibitors. Compound **30** was previously synthesized by their research group in a discovery campaign of FAAH inhibitors, but **30** selectively inhibited human ABHD6 (about 40% inhibition at 1 μM), without affecting FAAH or MAGL. An extensive structure–activity analysis led to the identification of the *meta*-amino analogue of the lead compound **30**, compound **31** (JZP-169, Figure 8), which exerted a notable AHBD6 inhibition with an IC_50_ value of 216 nM.^133^ The free amino group in the *meta* position seemed to be essential for the activity on ABHD6, since its protection or shift led to detrimental decreases of inhibition activity. Compound **31** was selective for ABHD6 when tested at 10 μM concentration, with any notable activity on other members of the ECS (FAAH, MAGL, ABHD12, and cannabinoid receptors). This novel and selective ABHD6 inhibitor interacts with the enzyme through an irreversible mechanism, as suggested by dilution assays and further confirmed by molecular docking studies. Docking of **31** underlined that the compound was located in the oxyanion hole, thus the carbonyl group of the inhibitor was suggested in proximity of the nucleophilic Ser148. Additionally, the importance of the free amino group on the benzyl moiety was explained by considering its involvement in hydrogen bonds with the side chains of Glu190 and Glu253.

1,2-Dihydro-2-oxo-pyridine-3-carboxamides were developed as potential CB2R ligands; however, this scaffold furnished a very promising ABHD6 inhibitor.^134^ 4-Methyl-5-bromo-2-substituted pyridine **32** (Figure 8) bound not only to both cannabinoid receptors as expected (*K*_i_ values of 113 and 606 nM for CB1R and CB2R, respectively) but exhibited a remarkable inhibition activity of ABHD6 enzyme with an IC_50_ value of 530 nM, exerting also inhibitory activity against anandamide cell uptake (IC_50_ = 620 nM), without affecting FAAH.

In 2021, a study about the role of 2-AG protection of the retina against the excitatory amino acid AMPA involved two ABHD6 inhibitors: AM12100 which was selective for ABHD6 (IC_50_ = 8 nM) and AM11920 which was a dual MAGL and ABHD6 inhibitor (IC_50_ values of 12.1 and 6.0 nM, respectively).^135^ The structures of both inhibitors are not disclosed yet. Interestingly, both compounds exerted a neuroprotective effect in the animal retinal model of AMPA excitotoxicity, but the selective ABHD6 inhibitor was less effective, thus leading to the conclusion that the dual inhibition exerted by AM11920 induced a more evident 2-AG increase and therefore it showed a better pharmacological profile.

It is noteworthy to add in this section a series of dual inhibitors of human ABHD6 and ABHD12 (ABHD12 will be analyzed in detail in section 10) discovered in 2014 by Kaczor et al. The authors screened an in-house library of heterocyclic compounds,^136^ leading to six weak inhibitors, pyrazole-based derivatives **33**–**37** (Figure 8) and thiosemicarbazide compound **38** (Figure 8). The remaining enzymatic activity on each enzyme was measured as a percentage compared to control and ranged from 65.3 to 84.2% for ABHD6 and 78.4 to 85.4% for ABHD12. Despite their low inhibition activity on both ABHDs, these heterocycles could represent a starting point for further structural modifications to tune their activity selectively on ABHD6 or ABHD12.

## 7 ABHD9

### 7.1 Biochemical Features and Biological Roles

ABHD9, also named epoxide hydrolase 3 (EPHX3), is a 360-amino acid protein (41 kDa) characterized by the presence of a nucleophilic aspartate in place of a serine. ABHD9 is prevalently expressed in skin and esophagus. ABHD9 was renamed EPHX3 after studies in which it displayed epoxide hydrolase activity against epoxyeicosatrienoic acids and 9,10-epoxyoctadecamonoenoic acids in vitro.^137^ Nevertheless, in a more recent in vivo study, genetic silencing of ABHD9 had no significant effects on the metabolism of fatty acid epoxides and did not alter LPS-induced lung inflammation or functional recovery after ischemia/reperfusion injury, that are two models regulated by epoxyeicosatrienoic acids.^138^ ABHD9-mediated hydrolysis of leukotoxin led to the production of a metabolite which was identified as a strong mediator of acute respiratory distress syndrome (ARDS).^137^ ABHD9 seems to be involved in cancer, since ABHD9 expression has been reported to be downregulated in tumors, such as prostate cancer,^139,140^ melanoma,^141^ B cell tumor,^142^ gastric cancer,^143^ salivary gland adenoid cystic carcinoma,^144^ oral squamous cell carcinoma,^145^ head and neck squamous cell carcinoma,^146^ and colorectal carcinoma.^147^ ABHD9 was considered a potential ichthyosis-related gene.^148^ The role of ABHD9 in the regulation of skin barrier function was confirmed by other studies; in fact, ABHD9 was found to be involved in the production of epidermis-related linoleate triols, considering that it is highly expressed in the external cells of human epidermis.^149^ Moreover, ABHD9 hydrolyzes linoleate-derived epoxy-alcohols esterified in skin ceramides in vivo.^150^

### 7.2 Inhibitors

**Figure 9:**
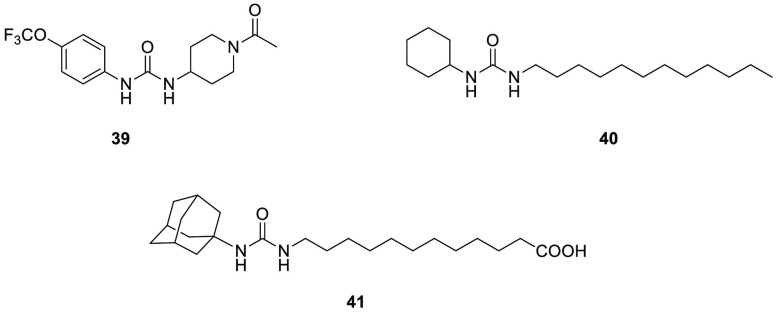
ABHD9 inhibitors.

In 2012 Decker and colleagues tested a class of *N*,*N*′-disubstituted urea derivatives, which previously were considered inhibitors of mammalian soluble epoxide hydrolase, on ABHD9.^137^ Among these *N*,*N*’-disubstituted urea derivatives, 1-(1-acetylpiperidin-4-yl)-3-(4-(trifluoromethoxy)phenyl)urea **39** (TPAU, Figure 9), 1-cyclohexyl-3-dodecylurea **40** (CDU, Figure 9), and 12-(3-adamantan-1-yl-ureido)-dodecanoic acid **41** (AUDA, Figure 9) were the most active inhibitors on ABHD9, with IC_50_ values of 75, 80, and 100 nM, respectively. These findings could be a starting point for the development of new ABHD9 inhibitors able to better elucidate their possible use as new therapeutic agents.

## 8 ABHD10

### 8.1 Biochemical Features and Biological Roles

ABHD10 is a 306-residue protein (34 kDa), ubiquitously expressed yet prevalent in kidney and thyroid. Proteomic studies located ABHD10 in the mitochondria.^151^ Some studies in the literature described the involvement of ABHD10 in drug metabolism.^152^ ABHD10 plays a key role in the metabolism of the immunosuppressant mycophenolate mofetil (MMF), because it led to the deglucuronidation in human liver of acyl glucuronide metabolite (AcMPAG), potentially responsible for some MMF-induced adverse effects such as leucopenia or gastrointestinal toxicity; therefore, ABHD10 exerted a detoxifying effect.^153^ A similar detoxifying activity was observed in the case of probenecid acyl glucuronide (PRAG), which is the main metabolite of the uricosuric agent probenecid, that can provoke severe allergic or anaphylactic reactions, as ABHD10 catalyzed PRAG deglucuronidation in human liver.^154^*S*-Depalmitoylase activity was observed for ABHD10; in particular ABHD10 acts on peroxiredoxin-5 (PRDX5), a key antioxidant protein and therefore ABHD10 can be included in the acyl protein thioesterases (APT) family of regulatory proteins.^155^

### 8.2 Inhibitors

**Figure 10:**
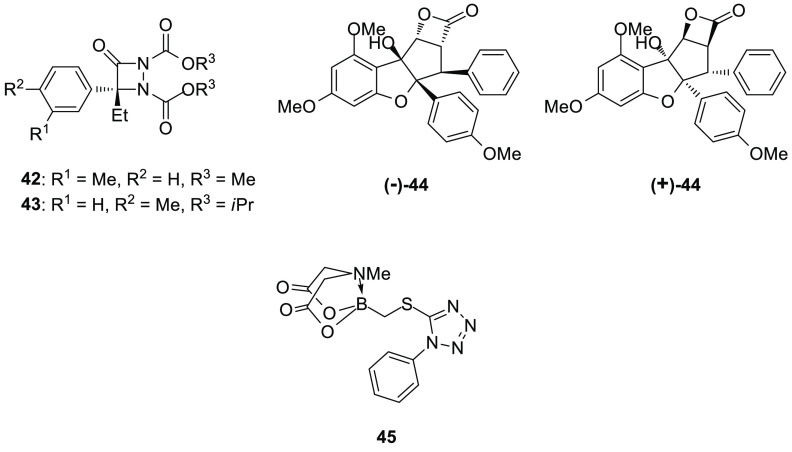
ABHD10 inhibitors.

Cravatt and his research group, with the aim to identify new serine hydrolase inhibitors, discovered a series of aza-β-lactams (ABLs), which efficiently inhibited the mammalian serine hydrolase protein-phosphatase methylesterase-1 (PME-1).^156^ Further structural optimization led to the identification of **42** (*R* enantiomer, ABL117, Figure 10), which inhibited both PME-1 and ABHD10 with IC_50_ values of 250 and 210 nM, respectively. Thereafter, in order to improve ABHD10 inhibition, the authors performed a SAR evaluation of the ABL scaffold. Using bulky substituents as *O*-alkyl groups on the carbamates or shifting the methyl group to the *para* position of the benzene ring increased potency for ABHD10, as demonstrated by compound **43** (*R* enantiomer, ABL303, also named ML257,^157,158^Figure 10), which showed an augmented inhibition potency on ABHD10 (IC_50_ = 30 nM) and a marked selectivity over ABHD6, prolyl endopeptidase (PREP), PME-1, and other serine hydrolases. Aza-β-lactam **43** maintained a notable activity in living Neuro-2a cells with an IC_50_ value of 21 nM, without exhibiting off-targets when tested at 1 μM concentration and no appreciable inhibition of PME-1 at 10 μM. The quantitative mass spectrometry-based proteomic method ABPP-SILAC was employed to further test **43** in Neuro-2a cells: it selectively and near-completely inhibited ABHD10 (>95%), and thus, **43** was the first discovered potent ABHD10 inhibitor. Compound **43** is an irreversible inhibitor, acting via aza-β-lactam ring opening and subsequent serine acylation.^157^

The same research group combined the flavagline rocaglate core, typical of natural compounds isolated from the genus *Aglaia*, characterized by a cyclopenta[*b*]-benzofuran structure, with a β-lactone scaffold, to give a class of rocaglate-derived β-lactones as potential serine hydrolases inhibitors.^159^ The most interesting derivative of this series is compound **44** (both enantiomers (+)-**44** and (−)-**44** are reported in Figure 10). Unfortunately, ABPP in proteomes deriving from human cancer cell lines (PC3 and LNCaP) and mouse tissues (brain, liver, and testes) and ABPP-SILAC analysis on PC3 cells pointed out that β-lactone **44** inhibited different serine hydrolases including not only ABHD10 (IC_50_ value of about 100 nM) but also cathepsin A (CTSA), retinoid-inducible serine carboxypeptidase 1 (SCPEP1), and acyl-CoA thioesterase 1/2 (ACOT1/2). In particular, the pure (−)-**44** enantiomer was shown to be responsible for most of the ABHD10 and ACOT1/2 inhibition activity in competitive APBB assay on PC3 cells. The authors hypothesized that compound (−)-**44** irreversibly inhibits the target hydrolase, by acylation of the active site nucleophilic serine, as has been reported for other β-lactones.

In 2012, Adachi et al. employed MIDA-boronates to identify new inhibitors of ABHD10 and serine carboxypeptidase (CPVL).^160^ Alkyl(MIDA)boronate **45** (Figure 10) was tested in ABPP experiments in PC3 cell proteome: its conversion into the corresponding boronic acid was evident in buffer after 2 h of incubation. Therefore, the inhibition required the decomposition of the (MIDA)boronate portion to the free boronic acid. Compound **45** induced a complete inhibition of ABHD10 with few off-targets and showed a near-complete inactivation of ABHD10 at 10 μM and of ACOT1/2 at 100 μM. CPVL inhibition was confirmed in ABPP-SILAC assays, in which **45** inhibited by more than 95% both ABHD10 and CPVL, at 25 μM.

## 9 ABHD11

### 9.1 Biochemical Features and Biological Roles

ABHD11 or Williams-Beuren syndrome chromosomal region 21 protein (WBSCR21) or PP1226 is a 315-amino acid protein (35 kDa). ABHD11 is a mitochondrial protein, mainly found in skeletal muscle,^161^ but it is an ubiquitous protein with higher expression in colon, prostate, small intestine, and thyroid. Its alternative name WBSCR21 originates from the fact that ABHD10 is among the deleted genes in Williams-Beuren syndrome, a severe neurodevelopmental disorder characterized by several diseases and abnormalities, concerning both physical and cognitive aspects.^162^ ABHD11 expression was reduced in white adipose tissue in mice fed with a high-fat diet as well as in HSL knockout mice. On the contrary, treatment with the antidiabetic drug rosiglitazone increased its expression; however, other analyzed lipases and esterases were unaffected. Therefore, the importance of these changes needs further elucidation.^163^ ABHD11 is involved in cancer aggressiveness, since increased ABHD11 is a predictive biomarker of metastases in lung adenocarcinoma.^164^ In breast cancer, ABHD11 was downregulated in paclitaxel-resistant MCF7/PacR cells (68% compared to MCF7 cells),^165^ but it was also related to breast cancer malignancy.^166^ Arya et al. expressed human ABHD11 in budding yeast, *Saccharomyces cerevisiae*, to further elucidate the role of this protein in lipid metabolism: ABHD11 overexpression decreased triacylglycerol content in yeast, thus playing a key role in lipid hydrolysis.^167^ ABHD11 involvement in the regulation of the metabolic state was confirmed by knockout ABHD11 mice, which did not gain weight when fed a high-fat diet, maintaining a lean phenotype, normal biochemical plasma parameters, and reduced fat intestinal absorption.^168^ ABHD11 regulates 2-oxoglutarate (2-OG) metabolism: genetic deletion of ABHD11 led to the accumulation of 2-OG, resulting in inhibition of 2-OG dependent dioxygenases which are involved in the hypoxia inducible factor (HIF) response, DNA methylation, and histone modifications. Moreover, ABHD11 proved to be fundamental for functional lipoylation of the 2-oxoglutarate dehydrogenase complex (OGDHc), the enzyme of the tricarboxylic acid cycle that decarboxylates 2-OG to succinyl-CoA.^169^ Recently, a role for ABHD11 in embryonic stem cell (ESC) maintenance was highlighted, determining that ABHD11 is important for self-renewal and metabolic homeostasis of ESC.^170^ The ABHD11 locus also encodes for long noncoding RNA, named ABHD11-antisense (ABHD11-AS1), whose increased expression was observed in gastric,^171^ colorectal,^172^ pancreatic,^173^ endometrial,^174^ nonsmall-cell lung,^175^ papillary thyroid,^176^ and ovarian cancer.^177^

### 9.2 Inhibitors

#### 9.2.1 Carbamate Derivatives

**Figure 11:**
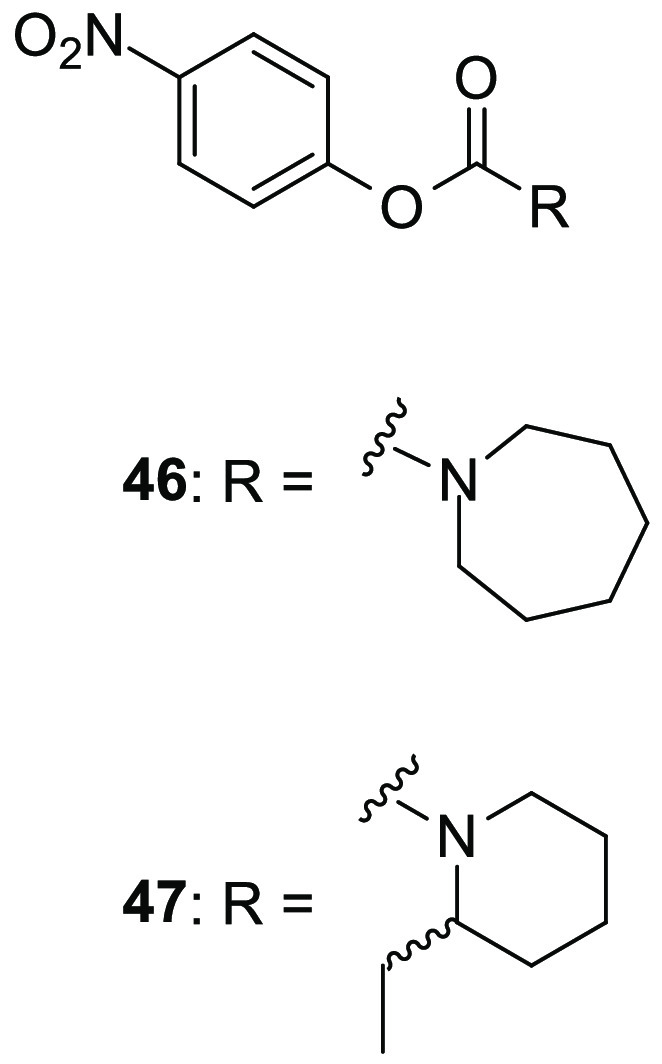
Carbamate-based ABHD11 inhibitors.

As mentioned before, the carbamate scaffold is very common among serine hydrolase inhibitors. During the screening study performed by Cravatt’s group to identify new inhibitors of serine hydrolases, during the discovery of compound **14**, compound **46** (WWL151, Figure 11) was identified as a mild inhibitor of ABHD11 (IC_50_ = 5.3 μM), however highly selective, likely due to the unicity of its seven-membered azepane ring, compared to other carbamate derivatives with broad spectrum activity on the panel of serine hydrolases. The substitution with a 2-ethylpiperidine ring proved to be successful, giving rise to a more potent inhibitor **47** (WWL222, Figure 11) which selectively blocked ABHD11 (IC_50_ = 170 nM) without any activity against other serine hydrolases.^115^ Carbamate **47** was also very efficacious and selective in vivo when administered intraperitoneally in mice at 10 mg/kg.

#### 9.2.2 Urea Derivatives

**Figure 12:**
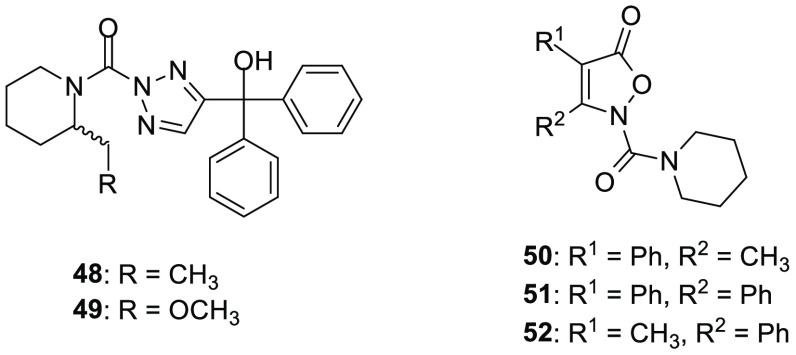
Urea-based ABHD11 inhibitors.

In 2010, Cravatt and colleagues carried out a fluorescence polarization-based competitive ABPP high throughput screening study to discover new inhibitors of lysophospholipase 1 and 2 (LYPLA1 and LYPLA2).^178^ During this study, performed on a library of triazole urea-based compounds, the authors serendipitously identified the racemic compound **48** (ML226, Figure 12) as a remarkably potent (IC_50_ = 15 nM) and selective (≥100-fold over more than 20 serine hydrolases) ABHD11 inhibitor, with a residual activity on *N*-acylaminoacyl-peptide hydrolase (APEH, 50% inhibition at 1.5 μM). The mode of action of **48** was assessed by LC-MS/MS studies, which revealed a covalent modification of the catalytic Ser141 of ABHD11, in which the triazole ring acts as the leaving group. A close analogue of **48**, derivative **49**(178) (AA44-2, Figure 12), bearing a bulkier methoxymethyl group instead of the ethyl group in 2-position of the piperidine ring, showed an improved ABHD11 inhibition with an IC_50_ value of 1 nM, still maintaining a high selectivity versus other serine hydrolases and no activity on APEH.^120^ These properties were confirmed by ABPP-SILAC analysis in living mouse T-cells: treatment with **49** resulted in a blockade greater than 95% of ABHD11 activity at the concentration of 3 nM with no cross-reactivity over other 40 serine hydrolases observed in T-cells.

In 2016, Navia-Paldanius and collaborators screened more than 200 in-house synthesized compounds designed to target serine hydrolases by using competitive ABPP tests.^179^ This screening led to the identification of three isoxazol-5(2*H*)-one-containing urea derivatives exerting a nanomolar potency against human ABHD11: **50** (JZP-228), **51** (JZP-245), and **52** (JZP-249) reported in Figure 12 showed IC_50_ values of 2.4, 3.4, and 2.3 nM, respectively. The three compounds were assessed in a competitive ABPP assay among the serine hydrolases of mouse whole brain membrane in order to evaluate their selectivity. All of them completely blocked ABHD11 activity when tested at 100 nM concentration; however, **50** inhibited an additional protein band, migrating at ∼60 kDa, attributable to FAAH. Moreover, **50** was previously found to be a HSL inhibitor with a reported IC_50_ value of 14 nM.^180^ The three inhibitors were tested in competitive ABPP with lysates of prostate cancer LNCaP and VCaP cells (both expressing FAAH) and PC3 cells (not expressing FAAH). Predictably, compounds **51** and **52** selectively blocked ABHD11, differently from **50** which confirmed its activity on FAAH in LNCaP and VCaP cells. Inhibitor **51** was further investigated in competitive ABPP: at 0.1 μM, **51** inhibited ABHD11 in all tested proteomes (mouse whole brain membranes, prostate cancer cell lysates, and mitochondrial fraction of brown fat and testicle), but at higher concentrations (1–10 μM) it also inhibited FAAH. Additionally, at 10 μM, **51** showed as off-targets ABHD6 and the serine hydrolase KIAA1363 in mouse whole brain membrane proteome. The cytotoxic effect of urea **51** was evaluated in prostate cancer cells: it reduced proliferation of the nonaggressive cell line LNCaP, but it was poorly effective on the aggressive cell line PC3. Nevertheless, in LNCaP and VCaP cells, **51** acted as a dual inhibitor targeting both ABHD11 and FAAH with a similar potency. Navia-Paldanius et al. built an ABHD11 homology model in order to better understand the interactions between these urea-based compounds in the enzyme active site. The docking studies suggested that the inhibitors properly fitted the active site of ABHD11, where they established π–π interactions. It was postulated a possible irreversible inhibition mechanism, through active site serine acylation, in which the isoxazol-5(2*H*)-one ring behaves as the leaving group.

## 10 ABHD12

### 10.1 Biochemical Features and Biological Roles

ABHD12 is also known as ABHD12A, c20orf22, or 2-arachidonoylglerol hydrolase, and it is a 398-residue protein (45 kDa). From a structural point of view, ABHD12 is a single-pass integral membrane protein, possessing a *N*-terminal transmembrane helix, which points its active site toward the extracellular space, and its catalytic triad is Ser246-Asp333-His372, as discovered by site-directed mutagenesis studies.^73,92^ The ubiquitously expressed ABHD12 has the highest expression in the brain (especially in microglia), and it is localized to the ER membrane in the mammalian brain,^181^ where it is responsible for about 9% of 2-AG hydrolysis, together with MAGL and ABHD6.^2^ ABHD12 is also present in macrophages and osteoclasts. Studies of substrate specificity revealed that ABHD12 prefers the 1(3)-isomer of arachidonoylglycerol over 2-AG and unsaturated C20:4 MAGs over C18:2 MAGs.^73^ It was found that ABHD12 required glycosylation for optimal activity and it showed a strong preference for very-long-chain lipid substrates, such as lysophosphatidylserine (lysoPS) lipids.^181^ Furthermore, in the brain, ABHD12 hydrolyzes oxidizedphosphatidylserine, which is considered an apoptotic signal, under severe inflammatory stress.^182^ Mutations of ABHD12 were found to be related to the etiology of some pathologies, such as the neurodegenerative disorder called polyneuropathy, hearing loss, ataxia, retinitis pigmentosa, and cataract “PHARC”, likely due to impaired 2-AG metabolism.^183,184^ Other studies suggest that PHARC may be induced by a dysregulated lysoPS lipase activity which is typical of ABHD12, since ABHD12 deficient mice displayed increased proinflammatory lysoPS lipid levels and neurobehavioral abnormalities similar to those of the PHARC phenotype.^185^ Together with ABHD16A, ABHD12 dynamically regulates lysoPS metabolism: ABHD16A contributes to the production of both cellular and secreted lysoPS starting from phosphatidylserine (PS), and ABHD12 preferentially controls degradation of secreted lysoPS to glycerophosphoserine, thus exerting complementary roles.^186,187^ A recent study in ABHD12 knockout mice reveals an upregulation of lipids deriving from arachidonic acid in the brain, thus suggesting that neuroinflammation may contribute to the development of PHARC-like symptoms.^188^ Dysfunctional ABHD12 has been linked to a variant of PHARC named Usher syndrome 3 (USH3), an autosomal recessive genetically heterogeneous disorder, characterized by congenital sensorineural hearing impairment and retinitis pigmentosa.^189,190^ Some tumor types showed an increased ABHD12 expression, such as in colorectal cancer^191^ and in breast cancer MCF7 and MDA-MB-231 cell lines ABHD12 knockdown reduced cell growth, proliferation, and invasiveness.^192^

### 10.2 Inhibitors

#### 10.2.1 Natural Compounds

**Figure 13:**
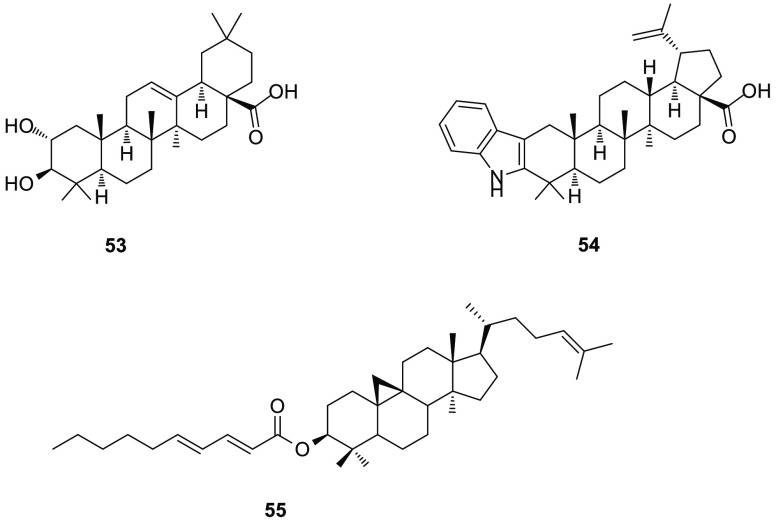
Natural ABHD12 inhibitors.

Encouraged by the fact that some natural triterpenes exerted a certain inhibition activity on hydrolases (i.e., MAGL and ABHD6), such as pristimerin **69**(193) (Figure 17), Parkkari et al. performed a screening of triterpene and triterpenoid derivatives by purchasing 15 commercially available compounds. The inhibition data were determined in lysates of HEK293 cells transiently overexpressing human ABHD12 and revealed that the oleanane derivative maslinic acid **53** (Figure 13) was the most potent ABHD12 inhibitor of this series, showing an IC_50_ value of 1.3 μM.^194^ A preliminary SAR study revealed that the presence of a carboxylate in position 17 in combination with small hydrophobic groups such as the methyl groups at position 4 determined a good inhibition activity. The screening of triterpene derivatives continued with a series of synthetic betulinic acid derivatives: among them, triterpene **54** (Figure 13), bearing an indole heterocycle fused with the central core in the place of the two hydroxyl groups of maslinic acid **53**, showed good inhibition of ABHD12 (IC_50_ = 0.9 μM). The authors enriched the SAR relative to this class of derivatives, since it was evident that the presence of hydrogen bond donors or acceptors at position 3 was required for an optimal inhibition activity. Later, the inhibition mechanism for the best two compounds was investigated: they proved to inhibit ABHD12 in a reversible manner, as tested by a dilution assay of the enzyme–inhibitor complex. Moreover, compounds **53** and **54** were tested in ABPP of HEK293 cell lysates and mouse brain membrane preparations and proved to be selective for ABHD12 over ABHD6, MAGL, FAAH, CB1R, and CB2R.

A cycloartane-type triterpene derivative **55** (Figure 13) isolated from *Euphorbia pterococca* proved to be a moderate ABHD12 inhibitor (IC_50_ = 11.6 μM); however, it was surprisingly selective, since it did not affect ABHD6, MAGL, and FAAH enzymes.^195^

#### 10.2.2 Synthetic Compounds

**Figure 14:**
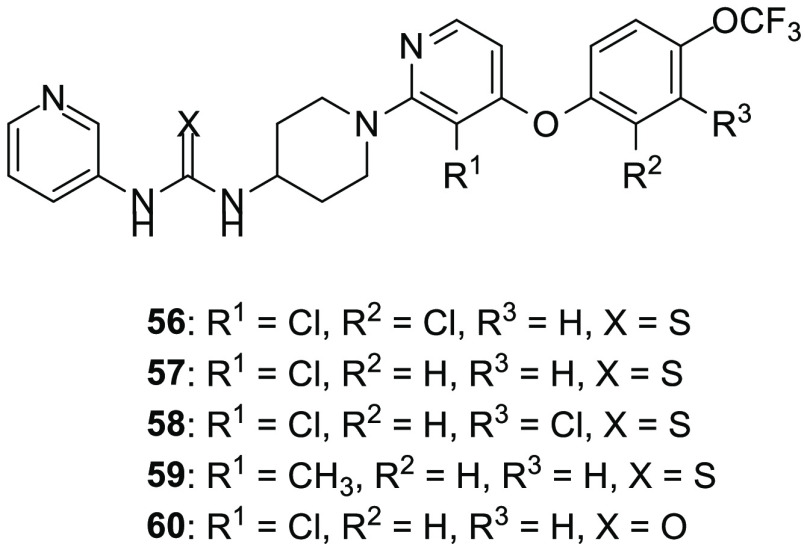
Synthetic ABHD12 inhibitors.

In 2019, the research group of Cravatt developed a thiourea derivative, **56** (DO264, Figure 14), which proved to efficiently and selectively inhibit ABHD12 in vitro and in vivo.^196,197^ An initial HTS procedure based on an innovative fluorescence assay, which measures the ABHD12-mediated hydrolysis of lysophosphatidic acid, was used to screen the Maybridge HitFinder library. Afterward, two further screenings, LC-MS-based lysoPS hydrolysis and ABPP assays, were pursued in order to identify new ABHD12 inhibitors and remove false positive compounds. After the identification of a hit compound based on a thiourea central core, a detailed SAR exploration of this scaffold led to the discovery of *N*-3-pyridyl-*N*′-4-piperidinylthiourea **56**, that competitively and selectively inhibited ABHD12, with and IC_50_ value of 11 nM in ABPP assays, in a competitive fashion, and in spite of its thiourea-based structure, **56** reversibly inhibited ABHD12. Compound **56** blocked lysoPS hydrolysis of recombinant mouse and human ABHD12 in transfected HEK293T cell lysates (IC_50_ values of about 30 and 90 nM against mouse and human ABHD12, respectively) and the lysoPS lipase activity of membrane lysates from mouse brain (IC_50_ = 2.8 nM) and human monocytic THP-1 cells (IC_50_ = 8.6 nM), confirming its activity. Considering that ABHD12 is highly expressed in macrophages, **56** was tested in THP-1 cells and primary human macrophages, in which it induced a significant increase in lysoPS and polyunsaturated 20:4 PS lipids. Inhibitor **56** provoked a high cytokine production in THP-1 cells; however, at concentrations of at least 5 μM, **56** exerted an undesired cytotoxic effect on this cell line. The authors excluded any cytotoxic effect generated by ABHD12 inhibition, since the block of the enzyme occurred at a lower concentration of the inhibitor, and this fact was confirmed testing **56** on different cell lines, when exposed to 1 μM **56**. An excellent ABHD12 inhibition was confirmed in C57BL/6 mice treated with **56** by intraperitoneal or oral administration, observing only a low inhibition of ABHD2 and phospholipase A2 group VI (PLA2G6). **56**-treated mice exhibited increased levels of brain lysoPS and 20:4 PS lipids, similarly to the changes observed in ABHD12 knockout mice, although they did not show any auditory defects, which are typical symptoms of PHARC disease. Moreover, **56**-treated and ABHD12 knockout mice exhibited a heightened immunological responses in a lymphocytic choriomeningitis virus (LCMV) clone 13 infection animal model, thus highlighting that ABHD12 may have an immunosuppressive function. A recently discovered effect of compound **56** is its ability to enhance ferroptotic death, a particular form of cell death defined by peroxidation of membrane phospholipids, in a similar fashion to what observed in ABHD12 knockout mice.^198^

Inhibitor **56** and structurally similar thiourea derivatives are also reported in a patent of the Scripps Research Institute, in which these ABHD12 inhibitors were claimed as useful for the treatment of cancer, neuropsychiatric disorders, and neurodegenerative, autoimmune, neuroinflammatory, and infectious diseases.^199^ Besides **56**, the most promising compounds are compounds **57**, **58**, and **59** (Figure 14), which displayed IC_50_ values lower than or equal to 100 nM for ABHD12 inhibition in competitive ABPP assays and in a substrate-based assay by using HEK293T cells overexpressing ABHD12.

In 2020, Lundbeck La Jolla Research Center Inc. published a patent including pyridinyl urea derivatives as ABHD12 inhibitors, which may be useful for the treatment of cancer and infectious, neurodegenerative, autoimmune, and neuroinflammatory diseases.^200^ The most potent inhibitor of this series was compound **60** (Figure 14), possessing the same structure of compound **57** (Figure 14) with the exception of the urea instead of the thiourea moiety. This pyridinyl urea inhibited mouse brain ABHD12 with an IC_50_ value lower than or equal to 100 nM, and it showed an ABHD12 inhibition activity greater than or equal to 75% when tested at 1 μM in mouse brain membrane proteomes.

## 11 ABHD16A

### 11.1 Biochemical Features and Biological Roles

ABHD16A is also named Human Lymphocyte Antigen B-associated transcript 5 (BAT5), and it is composed of 558 residues (63 kDa). It is a poorly known serine hydrolase, whose highest expression was observed in skeletal muscle and brain.^201^ ABHD16A is localized in the plasma membranes in human platelets and mouse megakaryocytes.^202^ It is palmitoylated; however, further investigation about this modification was not performed.^203^ The substrate preference for ABHD16A was investigated by Savinainen et al., and it was found that ABHD16A acts as a hydrolase for medium-chain saturated MAGs, long-chain unsaturated MAGs (in particular 1-linoleylglycerol, 1-LG) as well as the 15-deoxy-Δ^12,14^-prostaglandin J2-2-glycerol ester (15d-PGJ2-G).^204^ Polymorphisms of ABHD16A are correlated to Kawasaki syndrome, a disease characterized by vascular inflammation, which may cause coronary artery aneurysm formations and cardiac complications.^205^ In pigs, the polymorphism of ABHD16A was related with back fat thickness, thus suggesting its potential role as a marker associated with obesity.^206^ As already anticipated in subsection 9.1, ABHD16A is implicated in immunoregulation together with ABHD12, since both regulate lysoPS metabolism in vivo. In particular, ABHD16A regulates the lysoPS-induced release of proinflammatory cytokines from macrophages.^186^ The involvement of ABHD16 in immunoregulation originates from studies regarding its gene location, considering that ABHD16A belongs to a cluster of genes within the human major histocompatibility complex class III.^207,208^ Moreover, the expression of ABHD16A could influence the immunogenicity of bone marrow cells in mice.^209^

### 11.2 Inhibitors

**Figure 15:**
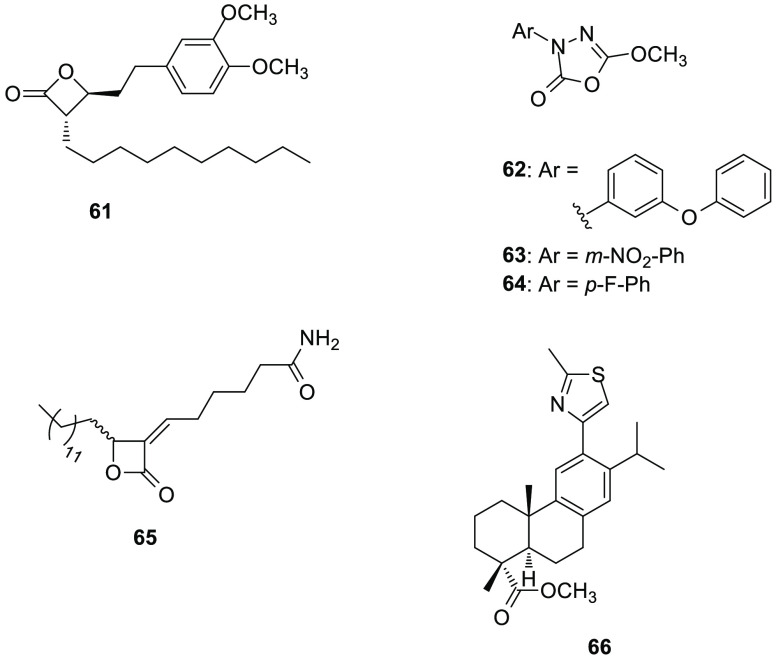
ABHD16A inhibitors.

In 2014, the first ABHD16A inhibitor was reported, the β-lactone palmostatin B **61** (Figure 15), which inhibited the hydrolysis of the MAG substrate 1-LG inhibitor in HEK293 lysates transfected with human ABHD16A, with an IC_50_ value of 100 nM.^204^ Considering thatpalmostatin B **61** was first developed as a LYPLA1 inhibitor (IC_50_ = 670 nM),^210^ further assays to determine its selectivity disclosed that compound **61** dose-dependently inactivated not only LYPLA1/2 and ABHD16A, but also ABHD12 (IC_50_ = 2 nM), ABHD6 (IC_50_ = 50 nM) and MAGL (IC_50_ = 90 nM). Considering the low selectivity of **61** and the potent activity of the HSL inhibitor **62** (C7600, Figure 15) on human ABHD16A (IC_50_ = 8.3 nM), Savinainen et al. decided to modify the 1,3,4-oxadiazol-2(3*H*)-one scaffold of **62** with the purpose to develop new selective ABHD16A inhibitors.^204^ Nevertheless, none of the modified derivatives of **62** showed an improved activity on the desired target. Two representative 1,3,4-oxadiazol-2(3*H*)-one derivatives are **63** and **64** (IC_50_ values of 63 and 32 nM for ABHD16A inhibition, respectively) reported in Figure 15. They differ from the lead compound because of the presence of a *m*-nitrophenyl (**63**) or a *p*-fluorophenyl ring (**64**) in the place of 3-phenoxyphenyl moiety of **62**. Competitive ABPP assays on mouse brain membrane proteome suggested that, at 1 μM concentration, 1,3,4-oxadiazol-2(3*H*)-ones **63** selectively inhibited ABHD16A with no cross-reactivity over other serine hydrolases such as FAAH, KIAA1363, MAGL, and LYPLA1/2 as they were off-targets of **62**, yet analogue **64** showed only activity against KIAA1363.

Considering the electrophilic nature of the β-lactone group, which is suitable to bind to the active site nucleophilic serine, a screening of α-alkylidene-β-lactone-based library of compounds^211^ led to the identification of derivative **65** (KC01, Figure 15) as an inhibitor of human and mouse ABHD16A, with IC_50_ values in the range 0.2–0.5 μM in competitive ABPP assays.^186^ A comparable inhibition activity was detected when a PS substrate was used in the membrane proteome of ABHD16A-transfected HEK293T cells, obtaining IC_50_ values of 90 and 520 nM for human and mouse ABHD16A, respectively. Further assays were performed in the membrane fraction of COLO205 (colon cancer), K562 (leukemia), and MCF7 (breast cancer) cancer cells, confirming the in situ inhibition of ABHD16A by **65**. Quantitative ABPP-MS experiments revealed that compound **65** also inhibited ABHD2 (94% at a concentration of 1 μM) as well as many other targets such as ABHD3, Patatin Like Phospholipase Domain Containing 4 (PNPLA4), PAFAH2, ABHD6, ABHD13, ABHD11, and LYPLA1 although to a minor extent (14–80%), thus revealing a low selectivity for ABHD16A. Furthermore, treatment of COLO205, K562, and MCF7 cells with **65** showed significant reductions in the levels of cellular lysoPSs compared to untreated cells, whereas different lipids were unaffected. Importantly, pretreatment of macrophages with **65** reduced the lysoPS-induced cytokine release, thus affecting immune response. Additionally, inhibitor **65** lowered the elevated lysoPS secretion observed in ABHD12-null cells derived from a PHARC subject. Compound **65** was also patented in 2016 by the Scripps Research Institute and the University of Connecticut.^212^ Pharmacological investigation of **65** suggested that ABHD16A plays a key role in the production of lysoPSs in both mammalian cells and in vivo and it should be a suitable target for the development of therapeutic agents to treat PHARC and other neuroinflammatory disorders.

The most recently discovered ABHD16A inhibitor is a diterpenoid of the abietane family, compound **66** (Figure 15), which was selected as the most promising inhibitor from a screening of an in-house library of 50 similar derivatives.^213^ Compound **66** led to a 23% remaining activity of ABHD16A when tested in lysates of HEK293 cells transfected with ABHD16A (IC_50_ value of 3.4 μM). It was selective over ABHD12 and demonstrated a reversible inhibition, as proved by dilution assays. Interestingly, the authors hypothesized allosteric binding of the compound because **66** reached an incomplete ABHD16A inhibition in all used assay conditions.

## 12 ABHD17A, ABHD17B, and ABHD17C

### 12.1 Biochemical Features and Biological Roles

Very little information is known about ABHD17 enzymes, which are broadly expressed in several cell types. They are localized in the plasma membrane and are identified as proteins able to depalmitoylate *N*-Ras.^214^ Palmitoylation is a biological process in which proteins are modified by the addition of palmitate, thus directing them to the cellular compartments where they play their specific functions. N-Ras is a protein that can promote the development of cancer, so the protein depalmitoylases ABHDs may influence tumor growth.

### 12.2 Inhibitors

**Figure 16:**
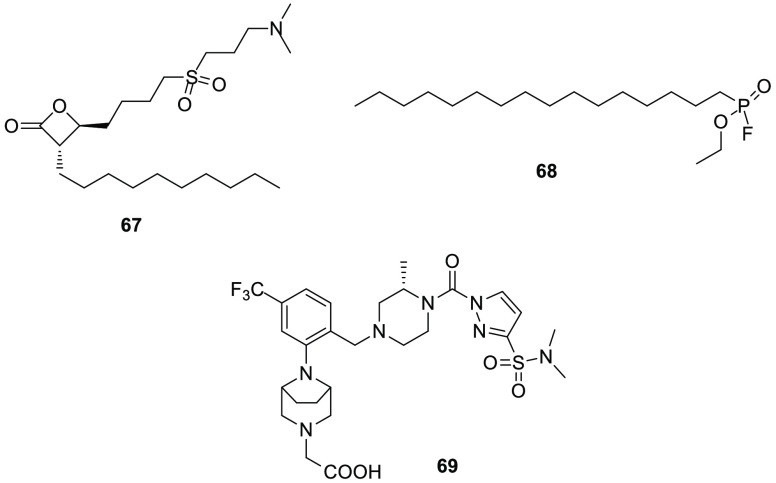
ABHD17 inhibitors.

Few inhibitors are reported in the literature for ABHD17 proteins, and among them the first discovered compounds are the unselective palmostatin B **61** (Figure 15),^210^ the analogue palmostatin M **67** (Figure 16),^215^ and hexadecylfluorophosphonate HDFP **68** (Figure 16).^216^ Palmostatin M **67** has the same β-lactone-based scaffold of palmostatin B, and it is metabolically unstable; moreover, it is unselective, since it also inhibits APT.^215^ Hexadecylfluorophosphonate HDFP **68** lacks selectivity, since it inhibits many different targets, above all fatty acid synthase (FASN), FAAH, neutral cholesterol ester hydrolase 1 (AADACL1), MAGL, and LYPLA1/2.^216^ In 2021, Remsberg et al. performed a gel-based ABPP screening in a native mouse brain proteome of a serine hydrolase-directed compound library developed at Lundbeck La Jolla Research Center, Inc., followed by a chemical optimization process aimed at improving selectivity and potency. This strategy led to the identification of a more selective pan-ABHD17 inhibitor ABD957 **69** (Figure 16).^217^ Compound **69** blocked covalently (likely due to its urea-containing moiety) more than 90% all the ABHD17 enzymes in MS-based ABPP experiments in OCI-AML3 (leukemia) cells, with a reported IC_50_ value against ABHD17B of 0.21 μM in lysates of HEK293T cells. Nevertheless, compound **69** cannot be defined as highly selective, due to a residual inhibition activity on CES1/2, ABHD6, and ABHD13. The importance of **69** derives from its contribution to the palmitoylation/depalmitoylation cycle, because it partially impaired N-Ras depalmitoylation in human acute myeloid leukemia (AML) cells, mainly affecting plasma membrane-associated dynamically palmitolylated proteins. Its therapeutic significance is linked to the impairment of NRAS mutant cancer cell growth, since the antiproliferative effect was abrogated in cells lacking ABHD17A and ABHD17B, thus confirming the ABHD17 inhibition by **69**.

## 13 Unselective ABHD Inhibitors

**Figure 17:**
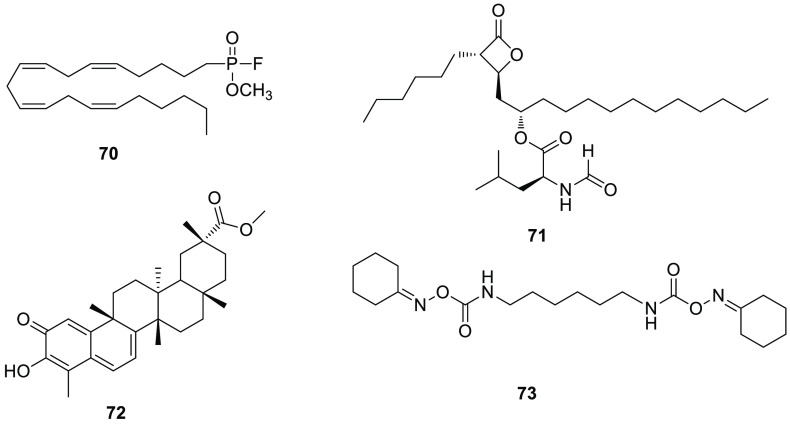
Unselective ABHDs inhibitors.

Some well-known nonselective ABHD inhibitors that deserve to be mentioned are included in this section. The most important are methylarachidonoyl fluorophosphonate (MAFP **70**, Figure 17), the approved antiobesity drug tetrahydrolipstatin (THL, Orlistat, **71** in Figure 17), the natural compound triterpene pristimerin **72** (Figure 17), and compound RHC-80267 **73** (Figure 17), and, with the exception of **71**, these compounds are generally used as pharmacological probes for biological studies rather than hit compounds to be developed as potential new drugs, because of their broad-spectrum activity. MAFP **70**, a FAAH inhibitor (IC_50_ = 2.5 nM),^218^ inhibited not only ABHD6 (IC_50_ = 0.017 μM) but also ABHD12 (IC_50_ = 0.087 μM)^73^ as well as other serine hydrolases (i.e., KIAA1363 and MAGL), and it was a cannabinoid receptor modulator. This compound was also reported in a patent dated 2017 concerning the progesterone activation of ABHD2.^219^ Treatment with compound **70** completely removed progesterone-dependent activation of calcium channel CatSper, without exerting any effect on basal CatSper activity. Moreover, ABHD2 inhibition by **70** blocked progesterone-activated calcium influx into sperm flagella. In this patent, it was demonstrated that the ABHD2 enzyme played a major role in mouse AR finely tuning intracellular calcium influx in mouse sperm. Compounds **71** and **73** were originally developed as DAGL-α/β inhibitors; however, they proved to be nonselective for these enzymes, targeting also other serine hydrolases.^220^ In fact, **71** inhibited ABHD6 (IC_50_ = 0.048 μM), ABHD12 (IC_50_ = 0.19 μM),^73^ and ABHD16A (IC_50_ = 0.03 μM).^220^ Derivative **70** inhibited ABHD6 (IC_50_ = 0.66 μM) and ABHD16A (IC_50_ = 23 μM).^220^ The reversible MAGL inhibitor (IC_50_ = 93 nM)^193^ pristimerin **72** blocked ABHD6 (IC_50_ = 1.3 μM^73^ or 98 nM,^193^ according to different experimental data reported in literature).

## 14 Conclusions and Future Perspectives

The family of serine hydrolases is one of the most diversified and numerous existing classes of enzymes. More than 200 enzymes belong to this class and all share a typical feature that is the presence of an active site serine, which is fundamental to catalyze the hydrolysis of substrates. Among serine hydrolases, ABHD proteins play many important roles in a wide range of pathophysiological processes and, thanks to their multifaceted roles, they have progressively acquired more importance in the scientific community. In this Perspective, the main members of the ABHD family are described for what concerns their biochemical role as well as their involvement in human diseases. Many ABHDs are involved in lipid metabolism, thus affecting obesity and fat-related diseases; moreover, some ABHD mutations are often correlated to rare genetic diseases. ABHD6 and ABHD12 are strictly connected to ECS since they are implicated in 2-AG metabolism. In addition, immunoregulation, cancer, and neurodegeneration are affected by downregulation or overexpression of ABHD enzymes.

Since 2007, many compounds targeting ABHD enzymes have been developed with the aim of blocking ABHD enzymes. Considering all the enzymes of the ABHD family, the research efforts led to a quite limited number of inhibitors, restricted to ABHD2, ABHD3, ABHD4, ABHD5, ABHD6, ABHD9, ABHD10, ABHD11, ABHD12, ABHD16A, and ABHD17A-C likely due to a scarce characterization of the remaining ABHD enzymes. An exception is represented by modulators of ABHD5 **7**–**9** which are efficient modulators of cellular lipolysis; however, they do not block ABHD catalytic activity but rather they are allosteric ABHD5 ligands. Future research lines should be aimed at characterizing the other less known members of the ABHD family, that are ABHD1, ABHD7, ABHD8, ABHD12B, ABHD13, ABHD14A, ABHD14B, ABHD15, ABHD16B, and ABHD18, to completely understand the complex biological roles of ABHD proteins. The identification of selective ligands for these proteins may help to fill a knowledge gap on endogenous lipid biosynthesis and metabolism as well as physiological and pathophysiological functions of these proteins in humans.

Other ABHD enzymes play key biological roles, although no selective ligands have yet been developed, and therefore, they are not extensively reviewed in this Perspective. For instance, ABHD1, whose biochemical function is not fully discovered, is ubiquitously expressed in murine and human tissues, reaching the highest expression in testis.^221^ ABHD1 overexpression was observed in a renal cell line: it contributed to the reduction of reactive oxygen species formation by NADPH oxidase, thus contributing to the protection against oxidative stress. For this reason, upregulation of ABHD1 in D5 dopamine receptor deficient mice, which develop hypertension and increased systemic oxidative stress, may contribute to the protective mechanism against oxidative stress.^222^

**Figure 18:**
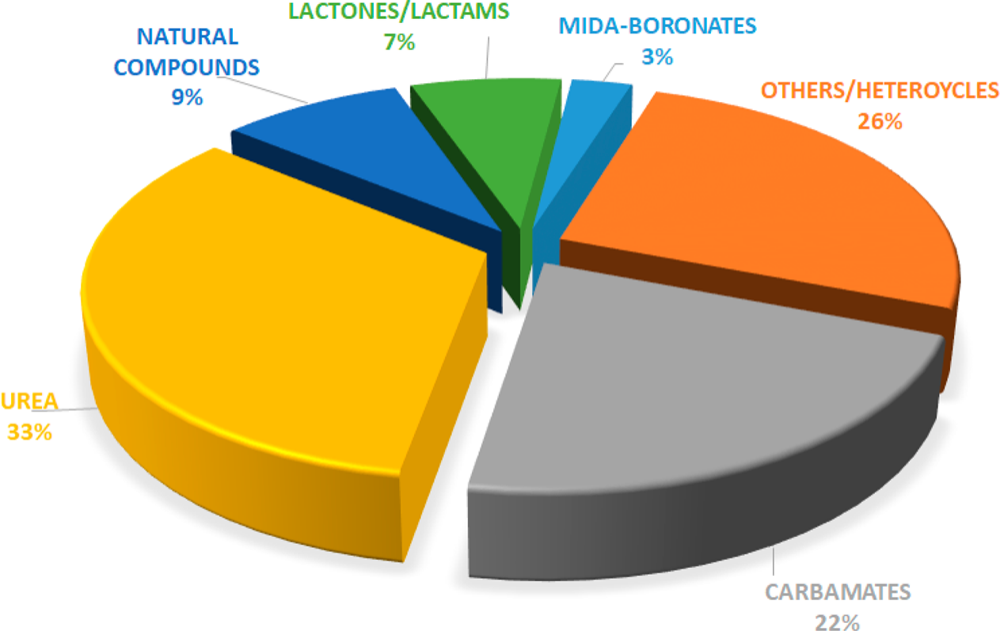
Percentages of the different chemical classes of ABHD targeting compounds, based on the representative ABHD inhibitors or modulators herein reported.

The herein reported ABHD-targeting compounds belong to several chemical classes (Figure 18). It is evident that most of them are urea- or carbamate-based molecules, since they exploit the intrinsic reactivity of active-site nucleophilic serine by promoting its acylation. Nevertheless, also lactones and lactams were expected to irreversibly bind to ABHD enzymes. Differently, compounds derived from natural sources, MIDA-boronates, and heterocycle-containing derivatives in general act as reversible inhibitors.

The structural similarity among ABHD proteins increases the risk of developing compounds inhibiting more ABHD enzymes, thus losing selectivity, and this may represent an obstacle to the development of an ideal clinical candidate. Nevertheless, considering the overlapping roles of some ABHD enzymes, the low selectivity of most ABHD inhibitors could be considered an opportunity to simultaneously interfere with a pathophysiological process by blocking different strategic targets.

In this Perspective, it is evident how many ligands were identified and profiled for their selectivity thanks to ABBP experiments. ABPP technology shows an undeniable utility in profiling ABHD inhibitor selectivity, because it allows one to determine the binding ability of a compound to different hydrolase enzymes at the same time in complex biological systems. The convenience and versatility of ABPP techniques were also exploited to identify potential off-targets, as it occurred for the FAAH inhibitor BIA 10-2474, which unfortunately provoked deleterious problems (death or neurological symptoms) in some volunteers during a phase 1 clinical trial. It was found that BIA 10-2474 and its main metabolite BIA 10-2639 inhibited many other enzymes, such as ABHD6 (inhibition greater than 90% at the tested concentrations) and ABHD11, in MS-based ABPP studies. The cross-reactivity with some serine hydrolases and the consequent marked alteration of the lipid metabolism may represent one of the causes of the compound’s neurotoxicity.^223^

The use of broad-spectrum probes in ABPP technology has accelerated the identification of new clinical candidates, finding new serine hydrolase inhibitors and assessing their selectivity both in vitro and in living systems, as in the case of ABHD inhibitors.^224^ The great improvement in the discovery of serine hydrolase inhibitors as new potential drugs made by application of ABPP assays is confirmed by two compounds which are currently studied in human clinical trials: MAGL inhibitor ABX-1431^225^ and FAAH inhibitor PF-04457845.^226^ In the future, further efforts should be directed toward the development of novel approaches enabling the identification of selective enzyme modulators, in order to speed up the drug discovery process. For example, the production of functional, pure recombinant ABHD enzymes and the development of reliable biochemical assays^227^ would be an important goal for a fast screening of large libraries of compounds aimed at finding new ABHD modulators.

## Abbreviations

AADACL1: neutral cholesterol ester hydrolase 1
ABHD: α/β-hydrolase domain
ABHD11-AS1: ABHD11-antisense
ABPP: activity-based protein profiling
AcMPAG: acyl glucuronide metabolite
ACOT1/2: acyl-CoA thioesterase 1/2
anandamide: AEA)*N*-arachidonoylethanolamine
2-AG: 2-arachidonoylglycerol
AMPARs: (RS-α-amino-3-hydroxy-5-methyl-4-isoxazolepropionic acid (AMPA)-type glutamate receptors
APEH: *N*-acylaminoacyl-peptide hydrolase
APT: acyl protein thioesterases
AR: acrosome reaction
ARDS: acute respiratory distress syndrome
ATGL: adipose triglyceride lipase
BMP: bis(monoacylglycero)phosphate
CB1R and CB2R: cannabinoid receptors 1 and 2
CES1: carboxylesterase 1
CES2: carboxylesterase 2
CoMFA: comparative molecular field analysis
COX-2: cyclooxygenase-2
CPVL: predicted serine carboxypeptidase
CTSA: cathepsin A
DAGL: diacylglycerol lipase
15d-PGJ2-G: 15-deoxy-Δ^12^,^14^-prostaglandin J2–2-glycerol ester
EAE: experimental autoimmune encephalomyelitis
EBV: Epstein–Barr virus
ECS: endocannabinoid system
EFT: Ewing family tumors
ER: endoplasmic reticulum
ESC: embryonic stem cell
FAAH: fatty acid amide hydrolase
FASN: fatty acid synthase
HCC: hepatocellular carcinoma
HDAC: histone deacetylase
HGSOC: high-grade serous ovarian cancer
HIC1: hypermethylated in cancer 1
HIF: hypoxia inducible factor
HSL: hormone-sensitive lipase
LCMV: lymphocytic choriomeningitis virus
1-LG: 1-linoleylglycerol
LPG: lysophosphatidylglycerol
LPI: lysophosphatidylinositol
Lp-PLA2: lipoprotein-associated phospholipase A2
LPS: lipopolysaccharide
LSD: lysosomal storage disorder
LYPLA1 and LYPLA2: lysophospholipase 1 and 2
lysoPS: lysophosphatidylserine
MAG: 1-monoacylglycerol
MAGL: monoacylglycerol lipase
MD: molecular dynamic
MEF2: myocyte enhancer factor-2
mGluR5: metabotropic glutamate receptor 5
MIDA: *N*-methyliminodiacetic acid
MMF: mycophenolate mofetil
mPGES-1/2: microsomial prostaglandin E synthase-1/2
mTORC1: mTOR complex 1
NAFLD: nonalcoholic fatty liver disease
NLSDI: neutral lipid storage disease with ichthyosis
NSCLC: nonsmall-cell lung carcinoma
2-OG: 2-oxoglutarate
OGDHc: 2-oxoglutarate dehydrogenase complex
PAFAH2: platelet activating factor acetylhydrolase 2
PC: phosphatidylcholine
PDAC: pancreatic ductal adenocarcinoma
PG: phosphatidylglycerol
PGD2-G: prostaglandin D2-glycerol ester
PGE2: prostaglandin E2
PHARC: polyneuropathy, hearing loss, ataxia, retinitis pigmentosa, and cataract
PLA2G6: phospholipase A2 group VI
PLA2G7: phospholipase A2 group VII
PLA2G15: lysosomal phospholipase A2 group XV
PLIN1: perilipin-1
PLIN5: perilipin-5
PME-1: protein-phosphatase methylesterase-1
PNPLA3: patatin like phospholipase domain containing 3
PNPLA4: patatin like phospholipase domain containing 4
PPARα and PPARγ: peroxisome proliferator-activated receptors α and γ
PRAG: probenecid acyl glucuronide
PRDX5: peroxiredoxin-5
PREP: prolyl endopeptidase
PS: phosphatidylserine
RA: retinoic acid
SAR: structure–activity relationship
SCPEP1: retinoid-inducible serine carboxypeptidase 1
SILAC: stable isotope labeling with amino acids in cell culture
TG: triacylglycerol
USH3: Usher syndrome 3
